# Global prevalence and risk factors of bovine tuberculosis in cattle: a systematic review and meta-analysis

**DOI:** 10.3389/fvets.2026.1820176

**Published:** 2026-04-22

**Authors:** Aboudou Habirou Kifouly, Orléanse Kouin, Géorcelin Alowanou, Obase Ngemani Bekindaka, Pierre Challaton, Abdou Satar Akadiri, Abdou Fadel Samba, John Dossou, Gilles-Christ Akakpo, Cyrille Kodoéito Boko

**Affiliations:** 1 Pan African University Life and Earth Sciences Institute (Including Health and Agriculture), University of Ibadan, Ibadan, Nigeria; 2 Laboratoire d’Ethnopharmacologie et de Santé Animale, Faculté des Sciences Agronomiques, Université d’Abomey-Calavi, Abomey-Calavi, Benin; 3 Immunology and Molecular Biology Laboratory of the Biotechnology Research Center, University of Yaoundé I, Yaoundé, Cameroon; 4 Unité de Biochimie et Substances Naturelles Bioactives (UBSNB), Laboratoire de Biologie Intégrative et d’Innovation Thérapeutique (BioInov), Université d’Abomey-Calavi, Abomey-Calavi, Benin; 5 Laboratoire de Biologie et de Typage moléculaire en Microbiologie, Université d’Abomey-Calavi, Abomey-Calavi, Benin; 6 Unité de Recherche sur les Maladies Transmissibles, Ecole Polytechnique d’Abomey-Calavi, University d’Abomey-Calavi, Abomey-Calavi, Benin

**Keywords:** bovine tuberculosis, meta-analysis, One Health, prevalence, risk factors, zoonosis

## Abstract

**Introduction:**

Bovine tuberculosis (bTB), caused by *Mycobacterium bovis*, is a chronic zoonotic disease of major global importance, particularly in low- and middle-income countries where surveillance and control remain inadequate.

**Objectives:**

This systematic review and meta-analysis aimed to estimate the global prevalence of bTB, identify key risk factors, and evaluate diagnostic approaches to support evidence-based control strategies within a One Health framework.

**Methods:**

A systematic search following PRISMA 2020 guidelines was conducted across eight electronic databases (PubMed, Web of Science, Scopus, ScienceDirect, Google Scholar, CAB Abstracts, SpringerLink, and AJOL) for studies published between 1990 and October 2025. A total of 7,775 records were identified, of which 98 studies met the inclusion criteria and were included in the quantitative synthesis.

**Results:**

The pooled global prevalence of bTB was estimated at 3.98% (95% CI, 3.94–4.03) and an overall herd’s estimate was 18.48% (95% CI, 17.47–19.49). Meta-analysis revealed a significantly lower odds of infection at the animal level compared to herd level (OR = 0.23; 95% CI: 0.15–0.37; *p* < 0.00001; I^2^ = 94%), indicating higher cumulative detection in herds. Subgroup analysis showed that Africa contributed the largest proportion of reported cases (61.22%), followed by Asia (30.61%), while other continents had marginal contributions. At regional level, Eastern Africa accounted for 41.84% of included studies, with Ethiopia representing the highest country-specific contribution (34.69%). Age-stratified analysis indicated higher prevalence in older cattle (≥5 years: 21.80%; 95% CI: 14.19–30.71) compared to younger animals (<5 years: 12.90%; 95% CI: 6.55–19.98), with borderline statistical significance (OR = 0.81; 95% CI: 0.66–1.00; *p* = 0.05). Among diagnostic methods, tuberculin-based skin tests were most frequently used (42.86%), followed by molecular techniques (23.47%). Sample type analysis showed the highest detection rates in skin samples (41.84%), followed by blood (28.57%) and milk (14.29%). Breed-specific analysis revealed higher prevalence in Holstein cattle (30.08%) compared to indigenous zebus (9.08%), suggesting increased susceptibility in improved breeds. Despite generally symmetrical funnel plots indicating low publication bias, substantial heterogeneity was observed across analyses (I^2^ > 75%), reflecting methodological and regional variability.

**Conclusion:**

Bovine tuberculosis remains a persistent and unevenly distributed zoonotic disease, with a disproportionate burden in Africa and Asia. The higher detection at herd level, significant influence of age and breed, and variability in diagnostic methods underscore the need for harmonized surveillance systems, improved diagnostic standardization, and targeted control strategies.

## 1 Introduction

Bovine tuberculosis (bTB), caused by *Mycobacterium bovis*, remains a major zoonotic disease of global concern, particularly in low- and middle-income countries where livestock production plays a critical role in food security and economic stability (1). As a member of the *Mycobacterium tuberculosis* complex (MTC), *M. bovis* infects a wide range of domestic and wild animal species and can be transmitted to humans, primarily through the consumption of unpasteurized dairy products or via direct contact with infected animals (2). In humans, the pathogen is mainly associated with extrapulmonary tuberculosis, whereas in cattle it causes a chronic granulomatous disease (3), characterized by progressive weight loss, lymphadenopathy, respiratory distress, and reduced productivity (4).

The global burden of bTB remains substantial despite long-standing eradication efforts in developed countries (5). It is estimated that over 50 million cattle are infected worldwide, with the highest prevalence reported in regions of Asia and Africa, where surveillance systems are often weak or fragmented (6). While countries such as Norway, Austria, and Canada have successfully implemented test-and-slaughter policies leading to near eradication (5), endemic persistence continues in many developing regions due to inadequate control programs, unrestricted animal movement, and limited veterinary infrastructure (7.3% prevalence in dairy herds in India) (7). Moreover, *M. bovis* exhibits a broad host range, infecting species such as buffaloes, goats, pigs, deer, badgers, and wildlife reservoirs, thereby complicating eradication efforts and facilitating cross-species transmission (4). Other MTC members (*M. caprae, M. orygis, M. microti, M. africanum*) also infect livestock and wildlife (8). Transmission occurs via aerosols, contaminated feed/water, or unpasteurized dairy (9), with rare vertical transmission through milk, colostrum, or in utero (10). Human infection arises from raw meat or direct contact during handling/slaughter (11).

Epidemiological evidence indicates considerable heterogeneity in reported prevalence rates across regions, production systems, and diagnostic methods. For instance, studies conducted in Bangladesh, report prevalence estimates ranging from low single digits (3.05–27.5%) depending on herd type (12), to over 25% (5.9–7.8%) (13) depending on the diagnostic tools employed, such as the tuberculin skin test (TST), ELISA, or molecular methods. In addition, *M. bovis* contributes variably to the human tuberculosis burden, accounting for up to 10–15% of new TB cases in developing countries, compared to less than 2% in industrialized nations. Such variability underscores the influence of methodological differences, ecological factors, and population characteristics on disease estimates. In Meta-analysis shows *M. bovis* accounts for 0.4–76.7% of human TB, pooled mean 12.1% (14), with 10–15% of new cases in developing countries versus 1–2% in industrialized nations (10). Cattle migration facilitates spread (15). In South Asia, crossbred, Holstein Friesian, and indigenous cattle are most affected (16).

Despite the growing body of literature on bovine tuberculosis, several important limitations persist in previous studies and reviews. First, many studies are geographically restricted and lack representativeness at regional or continental scales. Second, variations in diagnostic techniques and study designs have resulted in inconsistent and sometimes incomparable prevalence estimates. Third, existing reviews have often focused on specific countries, species, or diagnostic methods without providing a comprehensive synthesis integrating prevalence, risk factors, and diagnostic performance. Additionally, limited attention has been given to the heterogeneity of results and its underlying drivers, which restricts the generalizability and applicability of findings for policy and disease control strategies.

Given these gaps, there is a clear need for a robust and comprehensive systematic review and meta-analysis to synthesize available evidence on bovine tuberculosis. Such an approach allows for the generation of pooled prevalence estimates, the identification of major risk factors, and the evaluation of diagnostic methods while accounting for between-study variability. Importantly, this synthesis is essential to support evidence-based decision-making within a One Health framework, which recognizes the interconnectedness of human, animal, and environmental health in controlling zoonotic diseases such as bTB (17).

Therefore, the present study aims to (i) estimate the pooled prevalence of bovine tuberculosis in cattle and related livestock species, (ii) identify key epidemiological risk factors associated with infection, and (iii) assess the diagnostic methods used across different settings. We hypothesize that (1) the prevalence of bovine tuberculosis is significantly higher in developing regions compared to developed countries, (2) diagnostic methods significantly influence reported prevalence estimates, and (3) environmental, management, and host-related factors contribute substantially to disease heterogeneity. By addressing these objectives, this study seeks to provide a comprehensive and reliable evidence base to inform surveillance strategies, control policies, and future research directions.

## 2 Materials and methods

### 2.1 Study design and reporting framework

This study was designed as a systematic review and meta-analysis aimed at synthesizing evidence on the prevalence, risk factors, and diagnostic methods of bovine tuberculosis (bTB) in cattle. The review was conducted in accordance with the PRISMA 2020 (Preferred Reporting Items for Systematic Reviews and Meta-Analyses) guidelines to ensure transparency, reproducibility, and methodological rigor.

The study specifically focused on cattle (*Bos taurus* and *Bos indicus*) as the target animal species, excluding studies involving mixed animal populations without species-specific data.

#### 2.1.1 PICO framework

The eligibility criteria were defined using the PICO framework:

- Population (P): Cattle of any breed, age, or sex, raised under diverse ecological and geographical conditions worldwide.
- Intervention (I): Diagnostic approaches used for the detection of *Mycobacterium bovis*, including tuberculin skin tests (TST), enzyme-linked immunosorbent assay (ELISA), polymerase chain reaction (PCR), bacteriological culture, and interferon-gamma (IFN-*γ*) assays.
- Comparison (C): Comparisons were made across geographical regions, production systems (e.g., dairy, pastoral, mixed farming), animal management practices, specimen types, and diagnostic methods. Where applicable, comparisons were also conducted between infected and non-infected populations.
- Outcomes (O): The primary outcome was the prevalence of bovine tuberculosis. Secondary outcomes included the identification of associated risk factors (e.g., age, sex, breed, herd size, management system, wildlife contact) and the evaluation of diagnostic test performance (sensitivity, specificity, and field applicability).

### 2.2 Information sources and search strategy

A comprehensive and structured literature search was conducted in accordance with the PRISMA 2020 guidelines. Relevant studies published between January 1990 and October 2025 were systematically retrieved from multiple scientific databases, including PubMed, Google Scholar, Web of Science, Scopus, ScienceDirect, CAB Abstracts, SpringerLink, and African Journals Online.

Search strategies combined controlled vocabulary (e.g., MeSH terms) and free-text keywords using Boolean operators. A typical search query applied in PubMed was: (“*Mycobacterium bovis*” OR “bovine tuberculosis”) AND (“seroprevalence” OR “risk factors” OR “detection methods”) AND (“cattle” OR “bovine”) AND (“Continents”).

Database-specific adaptations of this strategy were applied. The complete search strings, execution dates, and the number of retrieved records from each database are provided in Supplementary File S1 to ensure reproducibility.

Additionally, reference lists of eligible studies were manually screened to identify further relevant publications.

### 2.3 Eligibility criteria

#### 2.3.1 Inclusion criteria

Studies were included if they:

- Were peer-reviewed full-text articles published in English or French.
- Employed observational study designs (cross-sectional, case–control, or cohort).
- Reported quantitative data on bovine tuberculosis prevalence, risk factors, or diagnostic methods.
- Included cattle populations only.
- Provided sufficient numerical data (sample size, number of positives, or prevalence estimates) to enable meta-analysis.
- Were conducted between January 1990 and October 2025.

#### 2.3.2 Exclusion criteria

Studies were excluded if they:

- Included multiple species without cattle-specific data.
- Were reviews, editorials, conference abstracts, or grey literature without primary data.
- Lacked extractable or reliable quantitative data.
- Fell outside the defined publication period.

### 2.4 Study selection process

All retrieved records were imported into Rayyan (https://www.rayyan.ai; version 2024) for screening and duplicate removal.

A two-stage screening process was conducted:

1. Title and abstract screening.
2. Full-text eligibility assessment.

Each stage was performed independently by two reviewers, with disagreements resolved through discussion or consultation with a third reviewer.

### 2.5 Data extraction procedure

A standardized and pre-tested data extraction form was used to ensure consistency. Two reviewers independently extracted data from each included study.

The following variables were collected:

- Author(s) and year of publication.
- Country and study region.
- Study design.
- Animal species (cattle), breed, age, and sex.
- Sample size and number of positive cases.
- Diagnostic methods used (e.g., TST, ELISA, PCR, culture).
- Type of biological samples.
- Reported prevalence and confidence intervals.
- Identified risk factors.

Where necessary, corresponding authors were contacted to obtain missing data. Graphical data were extracted using digital tools when numerical values were not explicitly reported.

### 2.6 Risk of bias and quality assessment

The methodological quality of included studies was assessed using the Joanna Briggs Institute (JBI) Critical Appraisal Checklist for Prevalence Studies, which is appropriate for observational epidemiological data (18).

Two independent reviewers conducted the assessment, and discrepancies were resolved by consensus. Studies were categorized as low, moderate, or high risk of bias based on predefined criteria.

### 2.7 Statistical analysis

#### 2.7.1 Meta-analysis model

Pooled prevalence estimates were calculated using a random-effects model (Der Simonian–Laird method) to account for between-study variability. Prevalence data were stabilized using the Freeman–Tukey double arcsine transformation prior to pooling.

#### 2.7.2 Effect measures

The primary outcome was pooled prevalence (%) with 95% confidence intervals (CI). Risk factor analyses were expressed using odds ratios (ORs) where applicable.

#### 2.7.3 Heterogeneity assessment

Statistical heterogeneity was evaluated using:

- Cochran’s Q test (*p* < 0.05 considered significant).
- I^2^ statistic, interpreted as:
- 25% = low heterogeneity.
- 50% = moderate heterogeneity.
- 75% and above = high heterogeneity.

#### 2.7.4 Forest plot analysis

Forest plots were generated to visually display individual study estimates and pooled prevalence values, including 95% confidence intervals. The size of each study’s weight was represented proportionally.

#### 2.7.5 Subgroup analysis

Subgroup analyses were conducted to explore sources of heterogeneity based on:

- Geographic regions.
- Diagnostic methods.
- Sample types.
- Production systems.

#### 2.7.6 Sensitivity analysis

A leave-one-out sensitivity analysis was performed to evaluate the robustness of pooled estimates by sequentially excluding each study.

#### 2.7.7 Publication bias

Publication bias was assessed using:

- Funnel plot symmetry.
- Egger’s regression test (19).

All statistical analyses were conducted using Review Manager (RevMan) version 5.4 and R statistical software (meta package).

### 2.8 Ethical considerations

This study was based exclusively on previously published data and did not involve direct animal experimentation. Therefore, ethical approval was not required. However, all included studies were verified to have obtained appropriate ethical clearance.

## 3 Results

### 3.1 Description of all included studies retrieved from electronics database and characteristics

**Figure 1:**
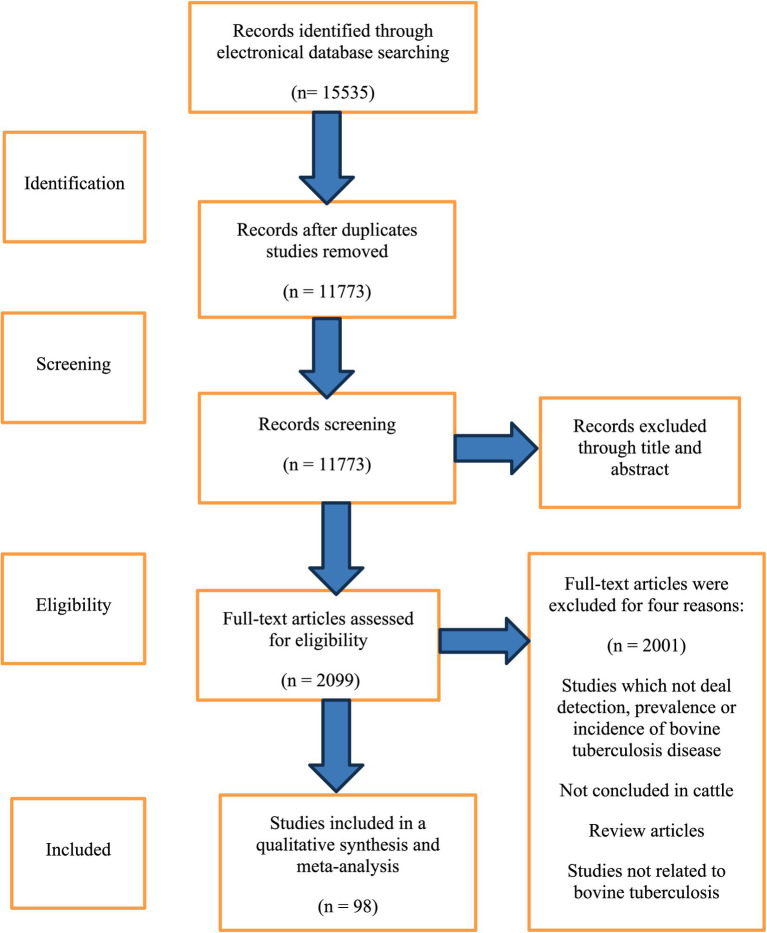
Flow chart of included studies based on PRISMA guidelines searching for the growth performance of chickens under heat stress in English and French.

Figure 1 and Table 1 summarize study characteristics per PRISMA. From 15,535 records, 11,773 were screened; 3,762 duplicates removed. Of 2,099 full texts, 98 met eligibility after excluding 2,001 (non-detection/prevalence/incidence, non-animal, reviews, unrelated). Studies spanned five continents, 29 countries, 13 regions. Only cattle were retained among species. Data on tested and positive bovine tuberculosis samples were extracted from all 98 studies.

**Table 1:** Characteristics of the data retrieved from all included studies.

ID	Authors and years	Country	Nature of animals	Sample	Positive	Sample_herds	Positive_herds
1	Abbate et al., 2020 (40)	Italy	Cattle	100,196	5,221	0	0
2	Adang et al., 2015 (41)	Nigeria	Cattle	320	85	0	0
3	Addo et al., 2016 (42)	Ghana	Cattle	94	6	0	0
4	Akinseye et al., 2017 (43)	Nigeria	Cattle	515	60	45	21
5	Aliraqi et al., 2020 (44)	Iraq	Cattle	106	20	0	0
6	Almaw et al., 2021 (131)	Ethiopia	Cattle	5,675	1776	0	0
7	Ambaw Endalew et al., 2017 (45)	Ethiopia	Cattle	720	119	0	0
8	Amemor et al., 2017 (26)	Ghana	Cattle	200	38	0	0
9	Ameni et al., 2007 (46)	Ethiopia	Cattle	5,424	732	0	0
10	Ameni et al., 2011 (47)	Ethiopia	Cattle	52	14	0	0
11	Ameni et al., 2016 (48)	Ethiopia	Cattle	1,171	548	12	11
12	Anne et al., 2017 (49)	India	Cattle	148	51	0	0
13	Asante-Poku et al., 2014 (50)	Ghana	Cattle	685	17	0	0
14	Ashenafi et al., 2013 (51)	Ethiopia	Cattle	110	15	0	0
15	Atik Faysal et al., 2025 (52)	Bangladesh	Cattle	500	53	0	0
16	Awah Ndukum et al., 2010 (53)	Cameroon	Cattle	385,784	3,163	0	0
17	Ayana et al., 2013 (54)	Ethiopia	Cattle	371	33	0	0
18	Aylate et al., 2013 (55)	Ethiopia	Cattle	1,029	63	0	0
19	Basit et al., 2015 (56)	Pakistan	cattle	200	15	0	0
20	Basit et al., 2018 (57)	Pakistan	Cattle	200	15	0	0
21	Belete et al., 2021 (58)	Ethiopia	Cattle	497	45	0	0
22	Biffa et al., 2010 (59)	Ethiopia	Cattle	3,322	117	0	0
23	Bonsu et al., 2000 (60)	Ghana	Cattle	1,200	166	0	0
24	Boukary et al., 2011 (61)	Niger	Cattle	393	14	0	0
25	Brown and de Anda, 1998 (62)	USA	Cattle	65,000	34	0	0
26	Buyuk et al., 2018 (63)	Turkey	Cattle	460	29	33	19
27	Cezar et al., 2016 (64)	Brazil	Cattle	401	8	20	6
28	Chakraborty et al., 2020 (65)	Bangladesh	Cattle	289	17	0	0
29	Cleaveland et al., 2005 (66)	Tanzania	Cattle	10,549	91	622	74
30	Cushicóndor-Collaguazo et al., 2023 (67)	Ecuador	Cattle	395	6	395	4
31	Das et al., 2018 (7)	India	Cattle	173	37	0	0
32	Dejene et al., 2016 (68)	Ethiopia	Cattle	2,550	141	0	0
33	Demelash et al., 2009 (69)	Ethiopia	Cattle	3,322	337	0	0
34	Dinka & Durassa, 2011 (70)	Ethiopia	Cattle	625	76	0	0
35	Duguma et al. 2017 (71)	Ethiopia	Cattle	554	21	0	0
36	Ejo et al., 2021 (72)	Ethiopia	Cattle	1,458	62	0	0
37	Elagdar et al., 2022 (73)	Egypt	Cattle	450	9	0	0
38	Ereqat et al., 2013 (74)	Palestinian	Cattle	208	4	0	0
39	Fetene & Kebede, 2009 (75)	Ethiopia	Cattle	453	44	75	41
40	Filia et al., 2016 (76)	India	Cattle	121	17	0	0
41	Firdessa et al., 2012 (77)	Ethiopia	Cattle	2,956	954	88	44
42	Ghumman et al., 2013 (78)	Pakistan	Cattle	9,021	1,057	0	0
43	Gogoi-Tiwari et al., 2022 (79)	Australia	Cattle	1,194	599	0	0
44	Gökmen et al., 2022 (80)	Turkey	Cattle	76	76	0	0
45	Gumi et al., 2012 (81)	Ethiopia	Cattle	207	48	0	0
46	Gumi et al. 2011 (82)	Ethiopia	Cattle	473	26	0	0
47	Gumi et al. 2012 (83)	Ethiopia	Cattle	421	10	0	0
48	Habarugira et al., 2014 (84)	Rwanda	Cattle	16,753	148	0	0
49	Habitu et al. 2019 (85)	Ethiopia	Cattle	1,357	59	0	0
50	Hassanain et al., 2009 (86)	Egypt	Cattle	46	38	0	0
51	Islam et al., 2020 (87)	Bangladesh	Cattle	1865	210	79	36
52	Islam et al., 2021 (88)	Bangladesh	Cattle	510	105	189	169
53	Kanyala et al., 2022 (89)	Burkina Faso	Cattle	2,165	58	0	0
54	Kapalamula, et al., 2023 (90)	Malawi	Cattle	1,547	112	0	0
55	Katale et al., 2013 (91)	Tanzania	cattle	1,103	27	0	0
56	Kazoora et al., 2014 (92)	Uganda	Cattle	525	11	63	7
57	Kemal et al., 2019 (93)	Ethiopia	Cattle	315	64	43	22
58	Khan et al., 2014 (94)	Pakistan	Cattle	30	4	0	0
59	Ullah et al., 2019 (95)	Pakistan	Cattle	2,400	141	0	0
60	Khan et al., 2024 (96)	Pakistan	Cattle	138	15	212	15
61	Koro Koro et al., 2013 (97)	Cameroon	Cattle	16,316	168	0	0
62	Kouengoua et al., 2024 (98)	Cameroon	Cattle	160	12	0	0
63	Leghari et al., 2016 (99)	Pakistan	Cattle	160	3	0	0
64	Leite et al., 2003 (100)	Brazil	Cattle	150	38	0	0
65	Lipiec et al., 2019 (101)	Poland	Cattle	32	32	0	0
66	Maqsood et al., 2024 (102)	Pakistan	Cattle	3,581	34	0	0
67	Mekibeb et al., 2013 (103)	Ethiopia	Cattle	500	25	0	0
68	Mekonnen et al., 2019 (104)	Ethiopia	Cattle	2,754	143	174	39
69	Mishra et al., 2005 (105)	India	Cattle	592	79	144	78
70	Mohammed et al., 2025 (132)	Ethiopia	Cattle	2,396	76	0	0
71	Moiane et al., 2014 (106)	Mozambique	Cattle	1,136	457	14	13
72	Mondal et al., 2014 (107)	Bangladesh	Cattle	101	6	56	4
73	Munyeme et al., 2010 (108)	Zambia	Cattle	944	64	111	13
74	Nega et al., 2012 (109)	Ethiopia	Cattle	311	22	77	11
75	Ntivuguruzwa et al., 2022 (110)	Rwanda	Cattle	300	165	70	39
76	Okeke et al., 2014 (111)	Nigeria	Cattle	168	36	0	0
77	Pandey et al., 2013 (112)	Zambia	Cattle	1,025	27	0	0
78	Rahman and Samad, 2008 (113)	Bangladesh	Cattle	47	15	0	0
79	Regassa et al., 2008 (114)	Ethiopia	Cattle	1,041	169	174	79
80	Sa’idu et al., 2015 (4)	Nigeria	Cattle	800	120	0	0
81	Shaltout et al., 2017 (115)	Egypt	Cattle	175	175	0	0
82	Shitaye et al.,2006 (116)	Ethiopia	Cattle	2098	392	0	0
83	Sichewo et al., 2019 (117)	South Africa	Cattle	129	11	0	0
84	Srivastava et al., 2008 (118)	India	Cattle	922	64	0	0
85	Thakur et al., 2010 (119)	India	Cattle	440	63	6	1
86	Thakur et al., 2012 (120)	India	Cattle	183	63	0	0
87	Tigre et al., 2012 (121)	Ethiopia	Cattle	384	82	0	0
88	Tipu et al., 2012 (122)	Pakistan	Cattle	1,000	454	0	0
89	Tora et al., 2022 (123)	Ethiopia	Cattle	221	18	0	0
90	Tschopp et al., 2009 (124)	Ethiopia	Cattle	2,216	111	450	56
91	Tsegaye et al., 2010 (125)	Ethiopia	Cattle	1,132	386	0	0
92	Tulu et al., 2021 (20)	Ethiopia	Cattle	654	257	0	0
93	Ullah et al., 2019 (95)	Pakistan	Cattle	1,146	79	2,259	141
94	Ullah et al., 2020 (126)	Pakistan	Cattle	2018	111	0	0
95	Wangmo et al., 2024 (127)	Bhutan	Cattle	971	25	0	0
96	Weldegebriel et al., 2025 (128)	Ethiopia	Cattle	380	22	30	8
97	Xu et al., 2021 (129)	China	Cattle	13,345	6,741	0	0
98	Zumárraga et al., 2012 (130)	Argentina	Cattle	0	0	257	102
	Total			**698,950**	**27,826**	**5,698**	**1,053**

This footnote was requested by reviewers and summarise total number of sample and positive sample.

### 3.2 Characteristics of diagnostics methods, specimens and quality assessment of all included studies

The Table 2 presents different characteristics of detection methods and the assessment of all included studies. Among 98 studies, 30 different diagnosis methods were used to detect bovine tuberculosis. Also, 04 different class groups were identified to be related to the objective of the farmers or breeders; such as: production, slaughtered, raising, and dairy. Concerning the type of specimen, six (06) different types of specimens were identified such as skin, blood, nasal discharges, milk, carcass and tissues. Related to the quality assessment of bias, 10 studies out 98 studies in general, were classified as “Strong studies” by following the different rules established by different tools used.

**Table 2:** Characteristics of diagnostics methods, specimens and quality assessment of all included studies.

ID	Authors and years	Country	Nature of animals	Group classes	Method used	Specimen	Quality Study Assessment
1	Mekonnen et al., 2019 (104)	Ethiopia	Cattle	Dairy	Single Intradermal Comparative Cervical Tuberculin (SICCT) test	Skin	Moderate
2	Cezar et al., 2016 (64)	Brazil	Cattle	Raising	Real-time quantitative PCR (qPCR)	Blood & milk	Moderate
3	Ashenafi et al., 2013 (51)	Ethiopia	Cattle	Raising	Single Comparative Intra-dermal Tuberculin (SCIDT) Tes	Skin	Moderate
4	Ejo et al., 2021 (72)	Ethiopia	Cattle	Slaughtered	MPT64 assay and Genotype line probe assay (LPA)	Carcass	Moderate
5	Kanyala et al., 2022 (89)	Burkina Faso	Cattle	Slaughtered	Line probe assays (LPA) GenoType Mycobacterium CM (common mycobacteria) and GenoType Mycobacterium MTBC mycobacterial growth indicator vials	Carcass	Moderate
6	Abbate et al., 2020 (40)	Italy	Cattle	Slaughtered	Histopathological methods	Carcass	Moderate
7	Adang et al., 2015 (41)	Nigeria	Cattle	Production	Tuberculin skin testing	Skin	Moderate
8	Addo et al., 2016 (42)	Ghana	Cattle	Production	Bovigam test	Blood	Moderate
9	Akinseye et al., 2017 (43)	Nigeria	Cattle	Production	Single intradermal comparative cervical tuberculin test	Skin	Moderate
10	Aliraqi et al., 2020 (44)	Iraq	Cattle	Raising	I-ELISA	Blood	
11	Almaw et al., 2021 (131)	Ethiopia	Cattle	Dairy	Comparative intradermal tuberculin skin testing and cultured	Milk	Moderate
12	Ambaw Endalew et al., 2017 (45)	Ethiopia	Cattle	Dairy	Comparative Intra-Dermal Tuberculin Test	Skin	Moderate
13	Amemor et al., 2017 (26)	Ghana	Cattle	Production	Ziehl- Neelsen staining while Anigen Rapid BTB Test was used for Cattle blood samples.	Blood	Moderate
14	Ameni et al., 2007 (46)	Ethiopia	Cattle	Production	Comparative intradermal tuberculin test	Skin	Moderate
15	Ameni et al., 2011 (47)	Ethiopia	Cattle	Production	GenoType Mycobacterium CM and AS	Skin	Moderate
16	Ameni et al., 2016 (48)	Ethiopia	Cattle	Dairy	Comparative intradermal tuberculin (CIT) test and bacteriologic	Milk	Moderate
17	Anne et al., 2017 (49)	India	Cattle	Production	PCR	Blood	Moderate
18	Asante-Poku et al., 2014 (50)	Ghana	Cattle	Dairy	Comparative intradermal tuberculin test	Skin	Moderate
19	Atik Faysal et al., 2025 (52)	Bangladesh	Cattle	Dairy	ELISA & PCR	Blood & Milk	Moderate
20	Awah Ndukum et al., 2010 (53)	Cameroon	Cattle	Production	Meat Inspection, Ziehl-Neelsen & Bacteria Culture	Carcass	Moderate
21	Ayana et al., 2013 (54)	Ethiopia	Cattle	Raising	Comparative intradermal tuberculin (CIDT) test		Moderate
22	Aylate et al., 2013 (55)	Ethiopia	Cattle	Slaughtered	Tuberculin test	Skin	Moderate
23	Basit et al., 2015 (56)	Pakistan	cattle	Production	Direct smear microscopy by Ziehl Neelsen & PCR	Tissue samples	Strong
24	Basit et al., 2018 (57)	Pakistan	Cattle	Dairy	PCR	Milk	Strong
25	Belete et al., 2021 (58)	Ethiopia	Cattle	Slaughtered	Smear microscopy and histopathology	Carcass	Moderate
26	Biffa et al., 2010 (59)	Ethiopia	Cattle	Production	Culture and microscopy (CM)	Carcass	Moderate
27	Bonsu et al., 2000 (60)	Ghana	Cattle	Production	Tuberculin test	Skin	Moderate
28	Boukary et al., 2011 (61)	Niger	Cattle	Production	Comparative Intradermal Tuberculin Test	Skin	Moderate
29	Brown et al., 1998 (62)	USA	Cattle	Slaughtered	Bacteriologic diagnosis	Carcass	Moderate
30	Buyuk et al., 2018 (63)	Turkey	Cattle	Production	ELISA	Blood	Moderate
31	Chakraborty et al., 2020 (65)	Bangladesh	Cattle	Raising	ELISA	Blood	Moderate
32	Cleaveland et al., 2005 (66)	Tanzania	Cattle	Production	Cattle interdermal test	Skin	Moderate
33	Cushicóndor-Collaguazo et al., 2023 (67)	Ecuador	Cattle	Production	Bacteriological culture	Lung level	Moderate
34	Das et al., 2018 (7)	India	Cattle	Production & dairy	Single intradermal tuberculin, comparative cervical tuberculin & Gamma interferon assay	Skin	Moderate
35	Dejene et al., 2016 (68)	Ethiopia	Cattle	Production	Comparative intradermal tuberculin test (CITT).	Skin	Moderate
36	Demelash et al., 2009 (69)	Ethiopia	Cattle	Slaughtered	Detailed meat inspection procedure.	Carcass	Moderate
37	Dinka & Durassa, 2011 (70)	Ethiopia	Cattle	Dairy	Single Intradermal Comparative Cervical Tuberculin (SICCT) test	Milk	Moderate
38	Duguma et al. 2017 (71)	Ethiopia	Cattle	Slaughtered	Smear microscopy and histopathology	Carcass	Moderate
39	Elagdar et al., 2022 (73)	Egypt	Cattle	Slaughtered	PCR	Blood & tissue	Moderate
40	Ereqat et al., 2013 (74)	Palestinian	Cattle	Slaughtered	PCR	Tissue & milk	Moderate
41	Fetene & Kebede, 2009 (75)	Ethiopia	Cattle	Production	Single comparative intradermal tuberculin test	Skin	Moderate
42	Filia et al., 2016 (76)	India	Cattle	Production	Single intradermal comparative cervical tuberculin (SICCT), bovine gamma-interferon (*γ*-IFN) & PCR		Strong
43	Firdessa et al., 2012 (77)	Ethiopia	Cattle	Dairy	Comparative intradermal tuberculin test (CIDT)	Skin	Strong
44	Ghumman et al., 2013 (78)	Pakistan	Cattle	Raising	Intradermal tuberculin test	Milk	Moderate
45	Gogoi-Tiwari et al., 2022 (79)	Australia	Cattle	Dairy	PCR & Mycoplasma immunogenic lipase A- Enzyme-Linked Immune Sorbent Assay (MilA ELISA).	Blood & nasal swab	Moderate
46	Gökmen et al., 2022 (80)	Turkey	Cattle	Slaughtered	PCR	Blood	Moderate
47	Gumi et al., 2012 (81)	Ethiopia	Cattle	Raising	CID-fast stained bacilli (AFB)	Carcass	Moderate
48	Gumi et al. 2011 (82)	Ethiopia	Cattle	Raising	CID-fast stained bacilli (AFB)	Carcass	Moderate
49	Gumi et al. 2012 (83)	Ethiopia	Cattle	Raising	Single Comparative Intra-dermal Tuberculin (SCIDT) Tes	Carcass	Moderate
50	Habarugira et al., 2014 (84)	Rwanda	Cattle	production	Microscopy with Kinyoun staining and isolation of mycobacterial species	Skin	Moderate
51	Habitu et al. 2019 (85)	Ethiopia	Cattle	Dairy	Comparative Intra-Dermal Tuberculin Test	Milk	Moderate
52	Hassanain et al., 2009 (86)	Egypt	Cattle	Dairy	Single intra dermal tuberculin test (SITT) & ELISA	Blood	Moderate
53	Islam et al., 2020 (87)	Bangladesh	Cattle	Production	Single intradermal comparative cervical tuberculin test (SICCTT)	Skin	Moderate
54	Islam et al., 2021 (88)	Bangladesh	Cattle	Production	Caudal fold (CFT) and comparative cervical tuberculin tests (CCT)	Skin	Strong
55	Kapalamula, et al., 2023 (90)	Malawi	Cattle	Slaughtered	MGIT, MTBC & Mpcr	Carcass	Moderate
56	Katale et al., 2013 (91)	Tanzania	Cattle	Production	Single Intradermal Comparative Tuberculin Test		Moderate
57	Kazoora et al., 2014 (92)	Uganda	Cattle	Production	Comparative intradermal tuberculin test (CIDT) test	Skin	Moderate
58	Kemal et al., 2019 (93)	Ethiopia	Cattle	Production	Comparative intradermal tuberculin skin test	Skin	Strong
59	Khan et al., 2014 (94)	Pakistan	Cattle	Raising	Culture, PCR, and Sanger sequencing	Blood	Moderate
60	Ullah et al., 2019 (95)	Pakistan	Cattle	Production	comparative cervical intradermal tuberculin test (CCIT)	Skin	Moderate
61	Khan et al., 2024 (96)	Pakistan	Cattle	Production	CCIT test & Ziehl Neelsen Stain	Skin	Moderate
62	Koro Koro et al., 2013 (97)	Cameroon	Cattle	Slaughtered	Ziehl-Neelsen staining	Skin	Moderate
63	Kouengoua et al., 2024 (98)	Cameroon	Cattle	Production	Comparative intradermal tuberculin test (CIDT) & simple intradermal tuberculin test (IDT)		Moderate
64	Leghari et al., 2016 (99)	Pakistan	Cattle	Dairy	Lowenstein-Jensen medium	Nasal discharge & milk	Moderate
65	Leite et al., 2003 (100)	Brazil	Cattle	Raising	classical biochemical tests, thin layer chromatography of mycolic acids and polymerase chain reaction-restriction fragment length polymorphism (PCR-RFLP) analysis	Blood & milk	Moderate
66	Lipiec et al., 2019 (101)	Poland	Cattle	Production	Initial tuberculin tests	Skin	Moderate
67	Maqsood et al., 2024 (102)	Pakistan	Cattle	Slaughtered	PCR	Blood	Moderate
68	Mekibeb et al., 2013 (103)	Ethiopia	Cattle	Slaughtered	mycobacteriological culturing, PCR	Skin	Moderate
69	Mishra et al., 2005 (105)	India	Cattle	Production	PCR-RFLP, N-PCR	Blood & milk	Moderate
70	Mohammed et al., 2025 (132)	Ethiopia	Cattle	Production	Single intradermal comparative cervical tuberculin test (SICCTT)	Blood sample	Moderate
71	Moiane et al., 2014 (106)	Mozambique	Cattle	Raising	Single comparative intradermal tuberculin test	Skin	Moderate
72	Mondal et al., 2014 (107)	Bangladesh	Cattle	Production	Anigen Rapid Bovine TB Ab test kit	Skin	Moderate
73	Munyeme et al., 2010 (108)	Zambia	Cattle	production	Comparative intradermal tuberculin test (CIDT) test	Skin	Moderate
74	Nega et al., 2012 (109)	Ethiopia	Cattle	Production	Comparative intradermal tuberculin test (CIDT) test	Blood	Moderate
75	Ntivuguruzwa et al., 2022 (110)	Rwanda	Cattle	Slaughtered	Culture, acid-fast bacteria staining, polymerase chain reaction, and GeneXpert assay	Blood	Moderate
76	Okeke et al., 2014 (111)	Nigeria	Cattle	Slaughtered	Ziehl-Neelsen test & duplex polymerase chain reaction technique (PCR)	Blood	Moderate
77	Pandey et al., 2013 (112)	Zambia	Cattle	Production	Single comparative intra-dermal tuberculin test	Skin	Moderate
78	Rahman and Samad, 2008 (113)	Bangladesh	Cattle	Production & dairy	Anigen Rapid Bovine TB Ab Test Kit	Blood	Moderate
79	Regassa et al., 2007 (114)	Ethiopia	Cattle	Production	Comparative intradermal cervical tuberculin test	Skin	Strong
80	Sa’idu et al., 2015 (4)	Nigeria	Cattle	Slaughtered	DNA-Based Polymerase Chain Reaction, Ziehl-Neelsen Techniques & DNA-Based Polymerase Chain Reaction	Blood	Strong
81	Shaltout et al., 2017 (115)	Egypt	Cattle	Production	Ziehl-Neelsen; Bacterial culture & PCR	Carcass & blood	Moderate
82	Shitaye et al.,2006 (116)	Ethiopia	Cattle	Slaughtered	Tuberculous lesions & Bovine tuberculin skin test	Carcass	Moderate
83	Sichewo et al., 2019 (117)	South Africa	Cattle	Production	PCR	Nasal swab & milk	Moderate
84	Srivastava et al., 2008 (118)	India	Cattle	Raising	Lowenstein-Jensen medium	Blood & milk	Moderate
85	Thakur et al., 2010 (119)	India	Cattle	Dairy	Tuberculin skin testing	Skin	Moderate
86	Thakur et al., 2012 (120)	India	Cattle	Dairy	PCR Restriction Analysis (PCR-RFLP) and Spoligotyping	Milk	Moderate
87	Tigre et al., 2012 (121)	Ethiopia	Cattle	Production	Comparative intradermal tuberculin test (CIDT) test	Blood	Moderate
88	Tipu et al., 2012 (122)	Pakistan	Cattle	Dairy	Modern PCR methods, Direct acid fast satining & Isolation	Milk	Moderate
89	Tora et al., 2022 (123)	Ethiopia	Cattle	Production	Comparative intradermal cervical tuberculin test	Skin	Strong
90	Tschopp et al.,2009 (124)	Ethiopia	Cattle	production	Comparative intradermal tuberculin	Skin	Moderate
91	Tsegaye et al., 2010 (125)	Ethiopia	Cattle	Dairy	Comparative Intra-Dermal Tuberculin Test	Milk	Moderate
92	Tulu et al., 2021 (20)	Ethiopia	Cattle	Dairy	Single intradermal comparative cervical tuberculin (SICCT) test	Skin	Moderate
93	Ullah et al., 2019 (95)	Pakistan	Cattle	Production	Comparative cervical intradermal tuberculin test		Moderate
94	Ullah et al., 2020 (126)	Pakistan	Cattle	Production & dairy	Culture and PCR	Skin	Moderate
95	Wangmo et al., 2024 (127)	Bhutan	Cattle	Production	bTB ELISA test kit.	Blood	Moderate
96	Weldegebriel et al., 2025 (128)	Ethiopia	Cattle	Dairy	Comparative intradermal tuberculin skin testing and cultured	Blood & milk	Strong
97	Xu et al., 2021 (129)	China	Cattle	Dairy	SIT, CIT, IFN-*γ* assay and ELISA	Blood	Moderate
98	Zumárraga et al., 2012 (130)	Argentina	Cattle bulk tank	Production	PCR	Blood	Moderate

### 3.3 Subgroups analysis of bovine tuberculosis by different geographical distribution of all included studies

#### 3.3.1 Continent and regions subgroup analysis

The overall pooled point estimate indicates a low aggregated prevalence of 3.98% (95% CI, 3.94–4.03) across 98 studies (with a total of 27,826 positive sample over 698,950 samples) and an overall herd’s estimate was 18.48% (95% CI, 17.47–19.49), suggesting substantial heterogeneity among included datasets (Table 3). Stratification by age group reveals a markedly higher prevalence in individuals aged ≥5–10 + years (21.8, 95% CI, 14.19–30.71) compared to those under 5 years (12.9, 95% CI, 6.55–19.98), indicating an age-associated increase in risk. At the continental level, Africa contributed the largest proportion of included studies (61.22%) with a relatively high pooled prevalence, followed by Asia (30.61%), while the Americas, Europe, and Oceania contributed minimally; however, the differences across continents were not statistically significant (*p* = 0.081). Regionally, significant heterogeneity was observed (*p* = 0.021), with Eastern Africa exhibiting the highest prevalence (41.84, 95% CI, 35.27–55.84) and the greatest associated risk ratio (RR = 10.51), followed by South Asia (25.51%, RR = 6.41) and Western Africa (10.20%, RR = 2.56), whereas other regions demonstrated comparatively lower prevalence estimates and risk ratios (≤4.08% and ≤1.03, respectively), often accompanied by wide confidence intervals, reflecting limited data and increased uncertainty. Overall, these findings highlight substantial geographic and age-related disparities in prevalence, alongside considerable between-study variability.

**Table 3:** Analysis by continent and region’s subgroups.

Category	No. of studies	Prevalence (%)	95% CI	*p*-value	Risk ratio
Overall point estimate	98	3.98	3.94–4.03	–	–
Overall herds estimate	98	18.48	17.47–19.49		
Age group					
<5 years	36	12.9	6.55–19.98	–	
≥5–10 + years	36	21.8	14.19–30.71	–	
Continent					
Africa	60	61.22	56.93–76.41	0.081	15.38
Asia	30	30.61	29.81–52.38		7.69
America	5	5.10	4.32–35.68		1.28
Europe	2	2.04	−3.15–23.15		0.51
Oceania	1	1.02	−3.99–12.68		0.26

Region	No. of studies	Prevalence (%)	95% CI	*P*-value	Risk ratio
Eastern Africa	41	41.84	35.27–55.84	0.021	10.51
Western Africa	10	10.20	5.81–21.59		2.56
South Asia	25	25.51	18.52–37.03		6.41
North Africa	3	3.06	−0.70–17.85		0.77
Southern Africa	3	3.06	−0.70–17.85		0.77
Central Africa	3	3.06	−0.70–17.87		0.77
Other regions (East Asia, Western Asia, Americas, Europe, Oceania)	≤4 each	≤4.08	Wide CIs		≤1.03

#### 3.3.2 Country subgroup analysis

The pooled prevalence varied markedly across countries (Table 4), with Ethiopia exhibiting the highest prevalence (34.69%) and a statistically significant association (*p* = 0.004), alongside the highest risk ratio (RR = 8.72), indicating a substantially elevated burden relative to the reference. Pakistan, India, and Bangladesh showed moderate prevalence estimates (11.22, 7.14, and 6.12%, respectively) with progressively decreasing risk ratios (2.82–1.54), suggesting intermediate endemicity levels. In contrast, Ghana and Nigeria demonstrated low and comparable prevalence (4.08%) with risk ratios close to unity (RR ≈ 1.03), indicating minimal relative risk differences. Cameroon and Egypt presented slightly lower prevalence (3.06%) with risk ratios below 1 (RR = 0.77), suggesting comparatively reduced risk. Countries with limited data (Tanzania, Rwanda, Brazil, Zambia, and others) showed very low pooled prevalence (≤2.04%) accompanied by wide or very wide confidence intervals, reflecting substantial uncertainty due to small sample sizes. Overall, the heterogeneity in prevalence and risk ratios underscores geographic variability and the influence of study representation on pooled estimates.

**Table 4:** Analysis by country subgroup.

Country	No. of studies	Prevalence (%)	95% CI	*P*-value	Risk ratio
Ethiopia	34	34.69	27.76–47.79	0.004	8.72
Pakistan	11	11.22	6.86–23.28		2.82
India	7	7.14	2.83–16.34		1.79
Bangladesh	6	6.12	1.92–14.52		1.54
Ghana	4	4.08	0.99–23.26		1.03
Nigeria	4	4.08	0.99–23.26		1.03
Cameroon	3	3.06	−0.70–17.85		0.77
Egypt	3	3.06	−0.70–17.85		0.77
Tanzania, Rwanda, Brazil, Zambia	2 each	2.04	Wide CIs		≤0.51
Other countries (single-study contributions)	1 each	1.02	Very wide CIs	–	0.26

### 3.4 Subgroups analysis of bovine tuberculosis by different groups of all included studies

#### 3.4.1 Diagnostic methods analysis subgroup

The subgroup analysis shows substantial variation in prevalence estimates and effect sizes across diagnostic techniques (Table 5). Tuberculin-based skin tests exhibited the highest pooled prevalence (42.86%; 95% CI: 36.36–56.97) and the strongest association (RR = 9.13; *p* = 0.015), indicating significantly higher detection compared to other methods. Molecular techniques (including PCR-based and sequencing approaches) yielded a moderate prevalence (23.47%; 95% CI: 20.85–42.16) with a considerable risk ratio (RR = 5.24), suggesting good diagnostic sensitivity but lower detection compared to skin tests. Microscopy and histopathological methods showed lower prevalence (10.20%; 95% CI: 5.81–21.59) with a modest risk ratio (RR = 1.46), reflecting limited sensitivity. Culture-based microbiological methods demonstrated relatively low prevalence (8.16, 95% CI: 3.79–18.12) and a risk ratio close to unity (RR = 0.95), indicating minimal differential detection. Immunological assays based on cytokine responses and serological tests both showed low prevalence estimates (3.06 and 8.16%, respectively), with wide confidence intervals and low risk ratios (RR = 0.11 and 0.95), suggesting limited and variable diagnostic performance. Genotypic assays produced a low to moderate prevalence (4.08, 95% CI: 0.99–23.26) with a low-risk ratio (RR = 0.25), indicating restricted but specific detection capability. Macroscopic/inspection-based methods had the lowest prevalence (2.04, 95% CI: −3.15–23.15) and the lowest risk ratio (RR = 0.07), reflecting poor sensitivity and high uncertainty.

**Table 5:** Diagnostic methods subgroups analysis.

Subgroup	Variants included	Effect size (*n*)	Prevalence (%)	95% CI	*P*-value	Risk ratio (RR)
Tuberculin-based skin tests	All intradermic forms	42	42.86	36.36–56.97	0.015	9.13
Molecular techniques	PCR, qPCR, PCR-RFLP, Spoligotyping, GeneXpert, Sanger sequencing	23	23.47	20.85–42.16		5.24
Microscopy & histopathology	ZN staining, Kinyoun stain, AFB smear, histopathology	10	10.20	5.81–21.59		1.46
Microbiological culture methods	LJ medium, MGIT, CM, biochemical tests + TLC	8	8.16	3.79–18.12		0.95
Immunological assays (cytokine-based)	IFN-γ assay, Bovigam, Gamma interferon	3	3.06	−0.44–8.66		0.11
Histopathology/Meat inspection	Tissue observation	3	3.06	−0.44–8.67		0.11
Genotypic assays	LPA, GenoType CM/AS/MTBC, MPT64	4	4.08	0.99–23.26		0.25
Macroscopic/inspection-based	Meat inspection, gross lesions	2	2.04	−3.15–23.15		0.07
Immunological assays (serology)	ELISA, I-ELISA, MilA, bTB ELISA	8	8.16	3.79–18.12		0.95

Overall, diagnostic method significantly influenced prevalence estimates (*p* = 0.015), with tuberculin-based tests yielding the highest detection rates, while microscopy, culture, and inspection-based approaches showed comparatively lower and less consistent detection performance.

#### 3.4.2 Specimen-based prevalence of bTB

The analysis shows that prevalence and risk of detection varied significantly across specimen types (*p* = 0.018) and animal class groups (*p* = 0.019) (Table 6). Among specimen types, skin samples exhibited the highest prevalence (41.84%; 95% CI: 35.27–55.84) and the highest risk ratio (RR = 8.86), indicating a substantially higher likelihood of detection compared to the reference category. This was followed by blood samples (28.57%; 95% CI: 21.55–40.68; RR = 5.41), milk samples (14.29%; 95% CI: 8.07–23.04; RR = 2.03), and carcass samples (13.27%; 95% CI: 7.18–21.71; RR = 1.80). Lower prevalence and negligible risk were observed for tissue samples (4.08%; 95% CI: 0.26–10.70; RR = 0.07) and nasal discharges (3.06%; 95% CI: −0.44–8.66; RR = 0.11), suggesting limited diagnostic yield from these specimen types.

**Table 6:** Specimen type and animal class group: subgroup analysis.

Specimen type	Effect size (*n*)	Prevalence (%)	95% CI	*P*-value	Risk ratio (RR)
Skin	41	41.84	35.27–55.84	0.018	8.86
Blood	28	28.57	21.55–40.68		5.41
Milk	14	14.29	8.07–23.04		2.03
Carcass	13	13.27	7.18–21.71		1.80
Tissue sample	4	4.08	0.26–10.70		0.07
Nasal discharges	3	3.06	−0.44–8.66		0.11

Production system (class group)	Effect size (*n*)	Prevalence (%)	95% CI	*P*-value	Risk ratio (RR)
Production animals	46	46.94	40.78–61.44	0.019	10.24
Dairy animals	26	26.53	19.52–38.25		4.90
Slaughtered animals	16	16.33	9.88–25.68		2.48
Raising animals	15	15.31	8.97–24.37		2.25

Regarding animal class groups, production animals had the highest prevalence (46.94%; 95% CI: 40.78–61.44) and the highest associated risk (RR = 10.24), indicating a markedly increased probability of positivity. This was followed by dairy animals (26.53%; 95% CI: 19.52–38.25; RR = 4.90), slaughtered animals (16.33%; 95% CI: 9.88–25.68; RR = 2.48), and raising animals (15.31%; 95% CI: 8.97–24.37; RR = 2.25), all showing progressively lower prevalence and risk.

Overall, the findings indicate that both specimen type and animal class significantly influence detection rates, with skin samples and production animals representing the highest-risk categories.

### 3.5 Meta-analysis of all animal and herds of included studies

**Figure 2:**
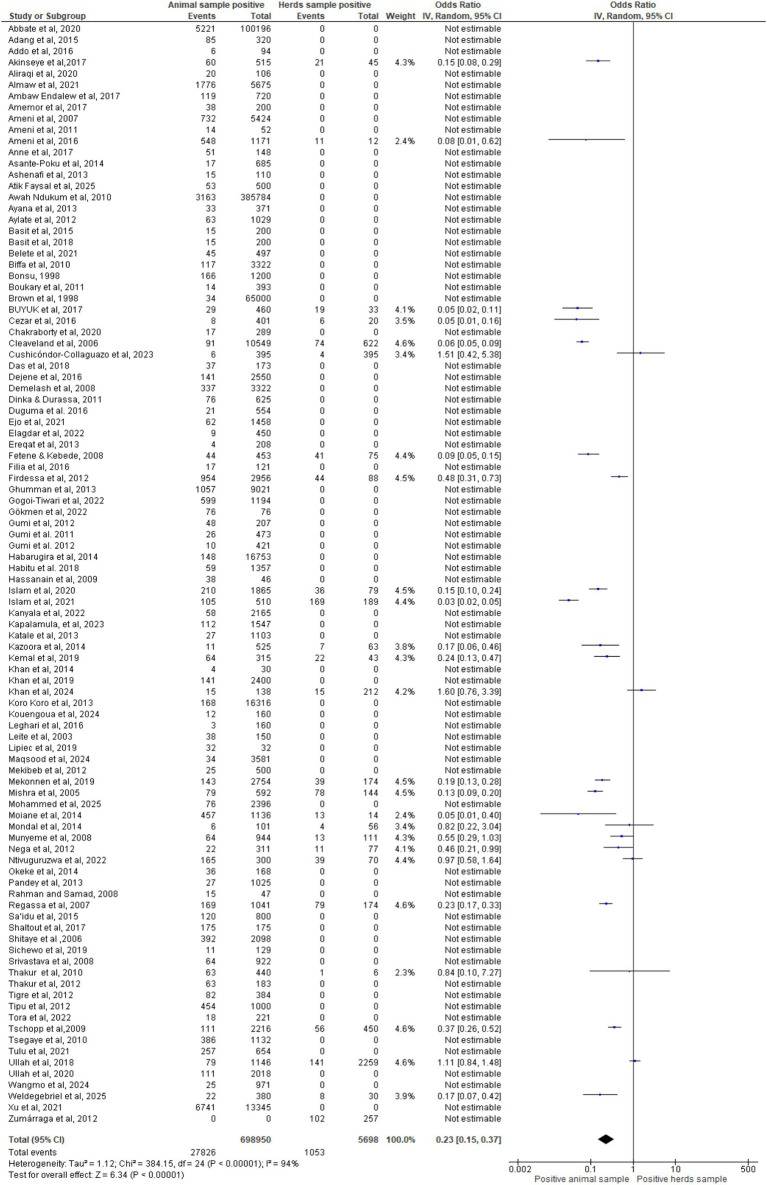
Forest plot of animals of included studies.

Figure 2 forest plot shows pooled OR = 0.23 (95% CI: 0.15–0.37, *p* < 0.00001), indicating markedly lower positivity in animals than herds. Heterogeneity was high (I^2^ = 94%, Tau^2^ = 1.12). Most studies reported OR < 1, consistent with reduced individual detection versus cumulative herd-level assessment. Overall effect was strong (Z = 6.34), though variability and non-estimable entries highlight the need for standardized protocols in bTB surveillance.

### 3.6 Publication of bias of included studies

**Figure 3:**
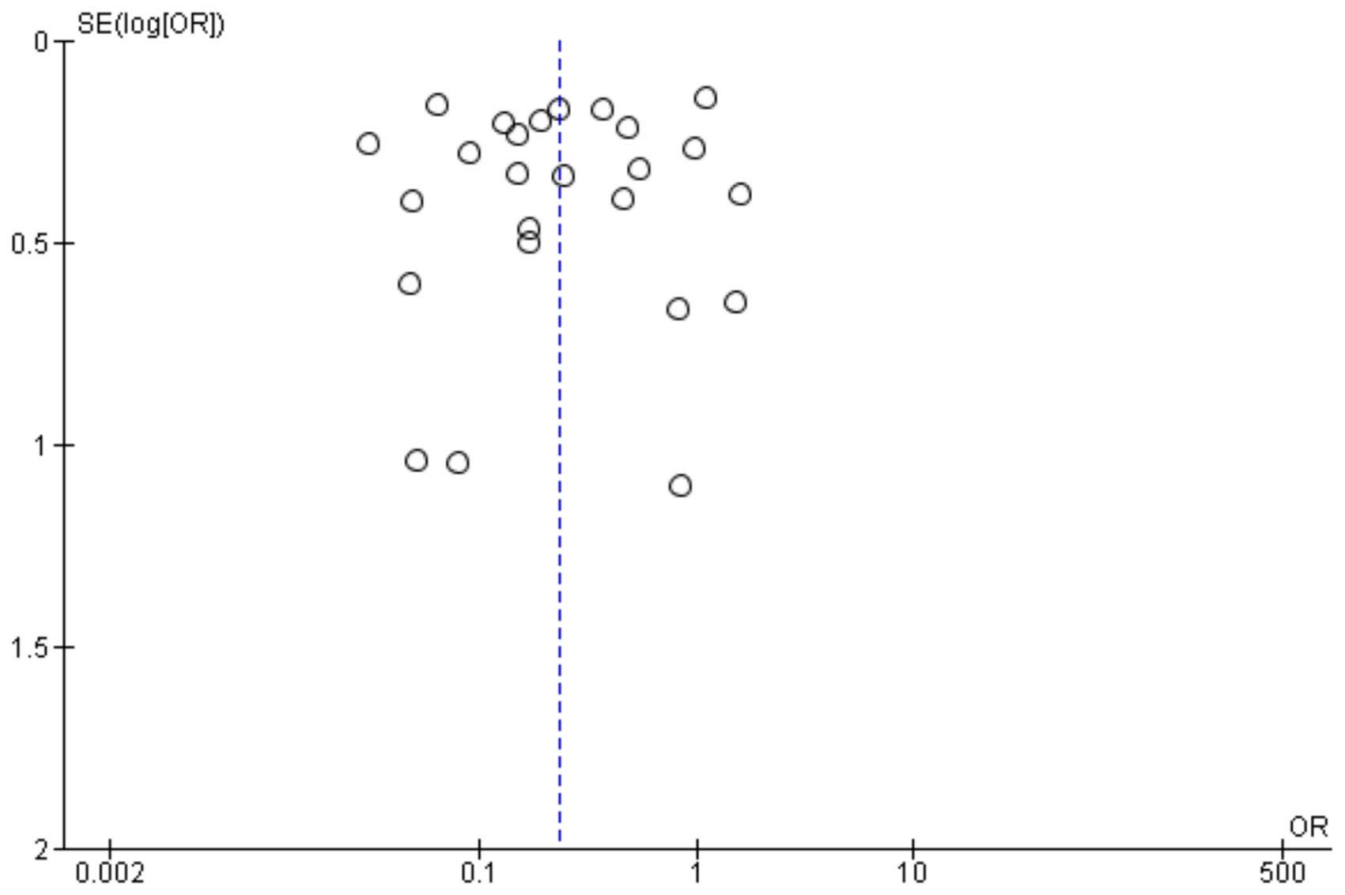
Funnel plot in general study.

Figure 3 shows a symmetrical funnel plot, indicating low risk of publication bias. Study dispersion follows expected precision gradients. The pooled OR (0.23) appears robust, though high heterogeneity suggests cautious interpretation across subgroups.

### 3.7 Meta-analysis of included studies by different continent

**Figure 4:**
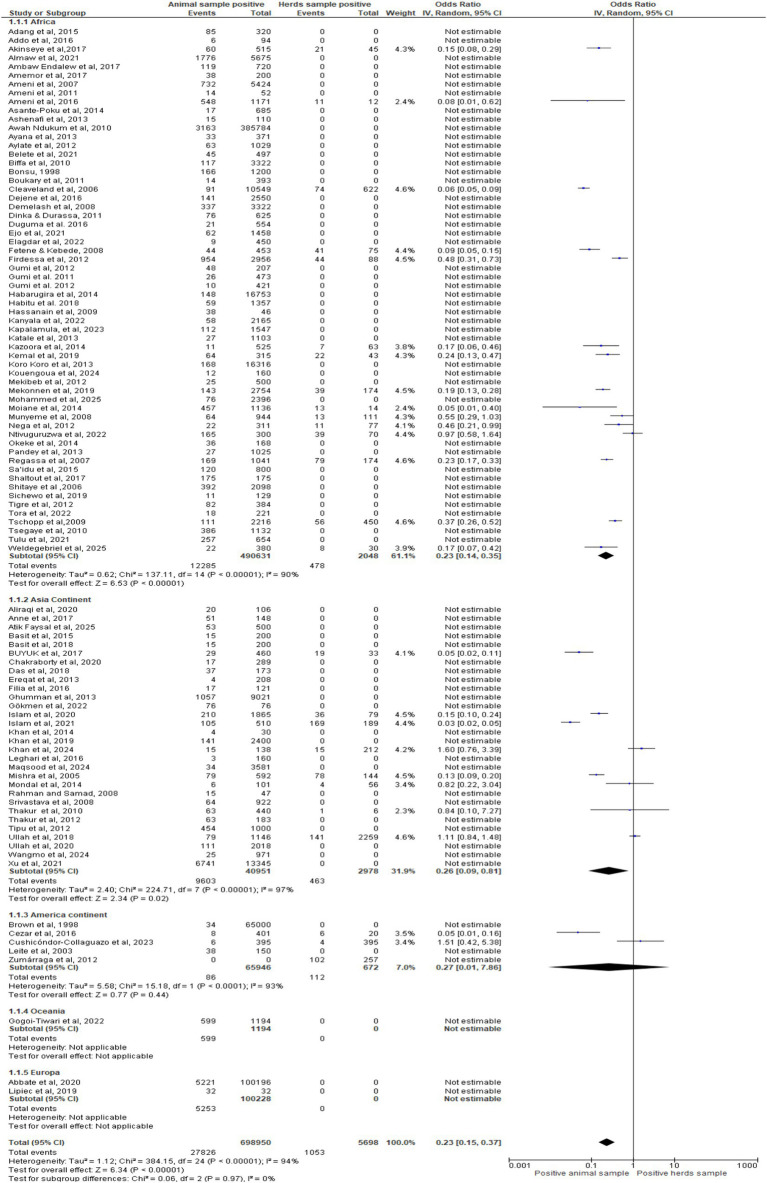
Forest plot by continents regions.

Figure 4 stratified meta-analysis shows regional disparities in bTB detection. Africa (OR = 0.23, Z = 5.63, *p* < 0.00001) and Asia (OR = 0.26, Z = 2.24, *p* = 0.02) confirm markedly lower positivity in animals versus herds, despite high heterogeneity (I^2^ = 90–97%). The Americas yield imprecise estimates (OR = 0.27 [0.01–7.86], *p* = 0.44), while Oceania and Europe lack pooled effects. Overall OR = 0.23 (95% CI: 0.15–0.37, Z = 6.34, *p* < 0.00001) underscores herd-level surveillance value and the need for harmonized diagnostic protocols.

### 3.8 Publication of bias of included studies by continent

**Figure 5:**
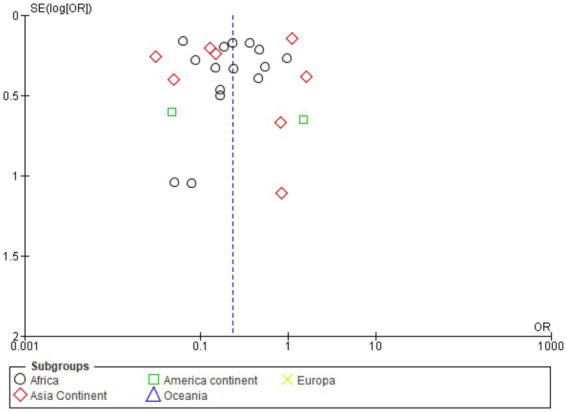
Funnel plot by continent.

Figure 5 funnel plot shows near-symmetry around OR = 1, indicating minimal publication bias. High-precision studies cluster at the top, with broader dispersion below, reflecting expected variance. African and Asian data dominate, consistent with epidemiological weight. Absence of marked asymmetry supports reliability, though high heterogeneity in forest plots cautions interpretation of regional variability.

### 3.9 Meta-analysis of included studies by different regions of continent

**Figure 6:**
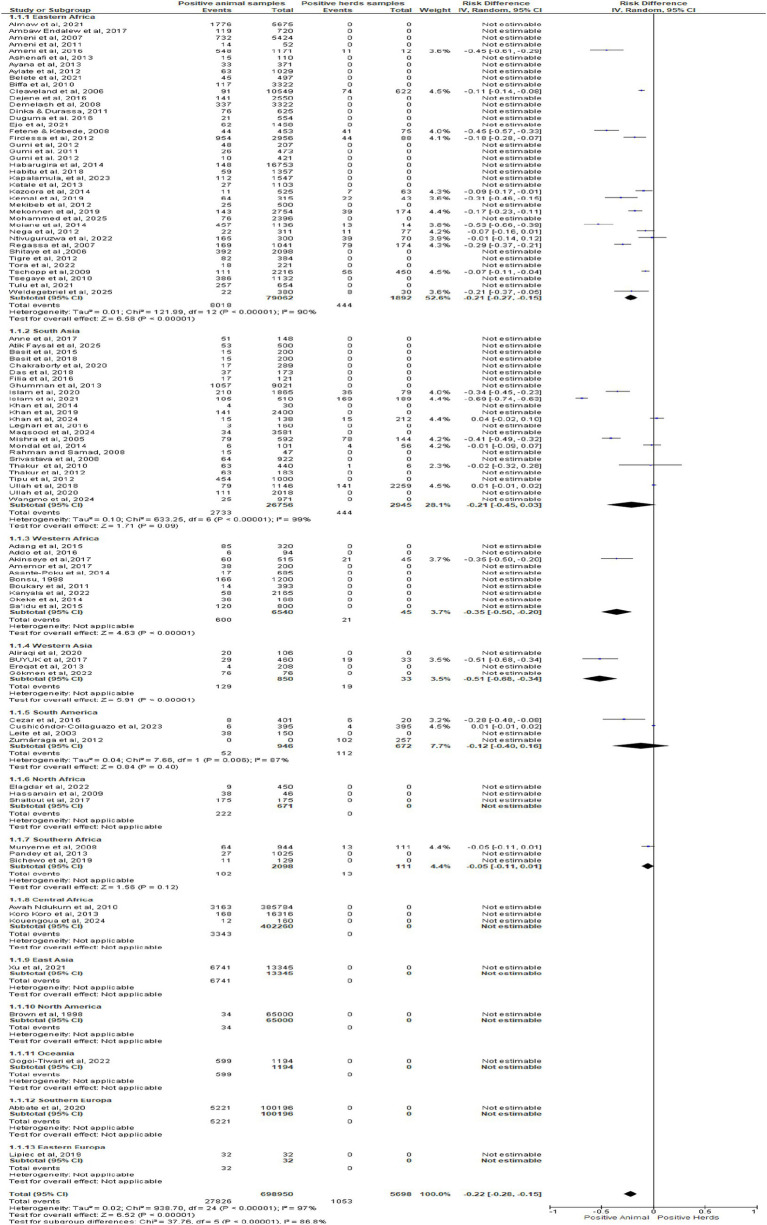
Forest plot of subgroups regions groups.

Figure 6 forest plot shows pooled risk difference −0.22 (95% CI: −0.28 to −0.15, Z = 6.52, *p* < 0.00001), confirming lower animal- versus herd-level positivity. Subgroup effects were marked in Eastern Africa (−0.21), Western Africa (−0.35), and Western Asia (−0.51). Heterogeneity was high (I^2^ = 97%), while several regions lacked estimable data. Results highlight herd-level surveillance advantage and the need for standardized diagnostic protocols.

### 3.10 Publication of bias of included studies by different regions of continent

**Figure 7:**
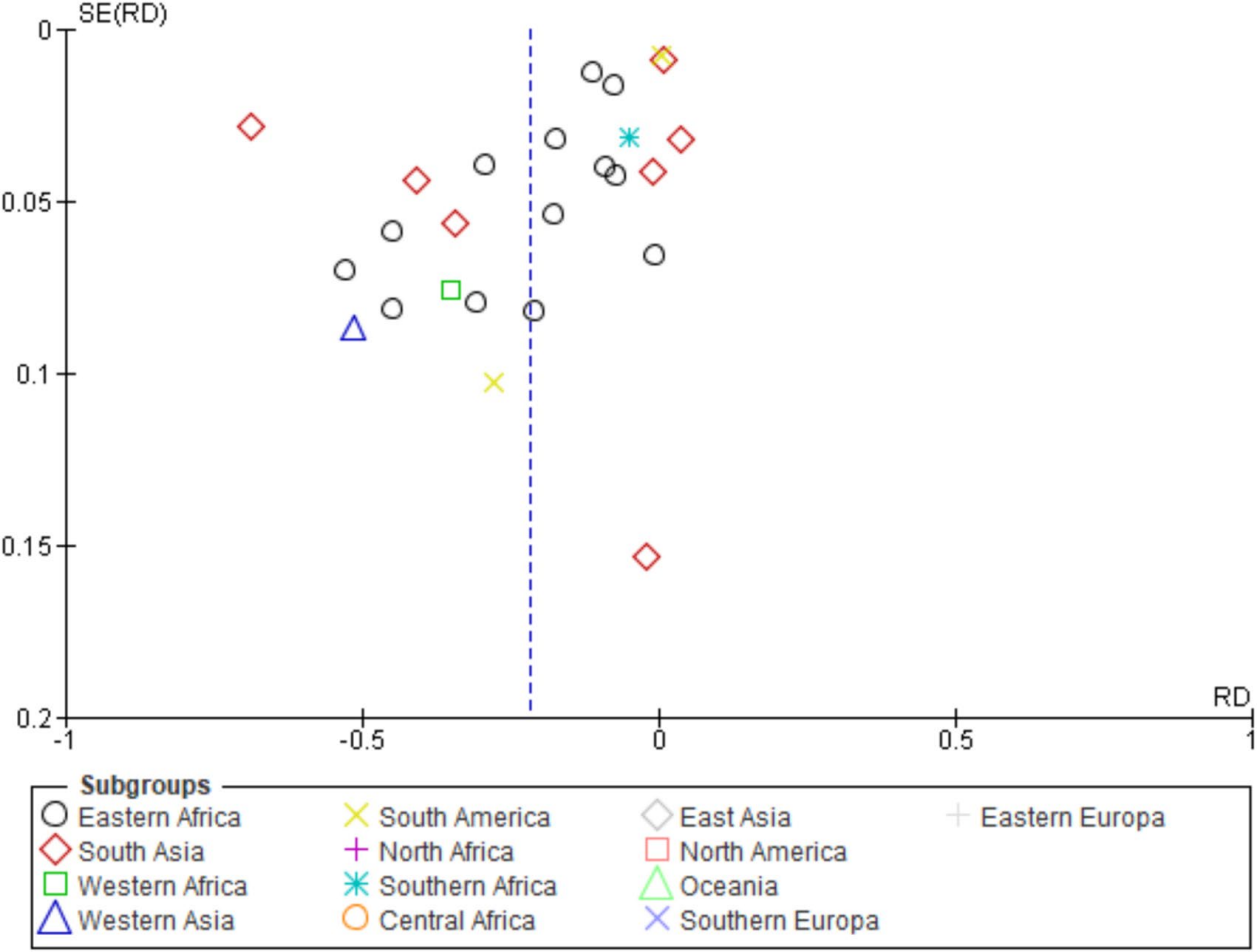
Funnel plot of included studies by different regions of continent.

Figure 7 scatter plot shows RD distribution and SE[RD] across subgroups. Symmetrical dispersion around RD = 0 indicates no publication bias; most points cluster at SE < 0.1, reflecting high precision. Eastern Africa, South Asia, and Western Africa display more negative RD, confirming lower animal- versus herd-level detection. North America, Oceania, and Europe show minimal deviation, likely due to limited data. Overall, results support pooled robustness while underscoring regional heterogeneity in diagnostic performance.

### 3.11 Meta-analysis of included studies by age

**Figure 8:**
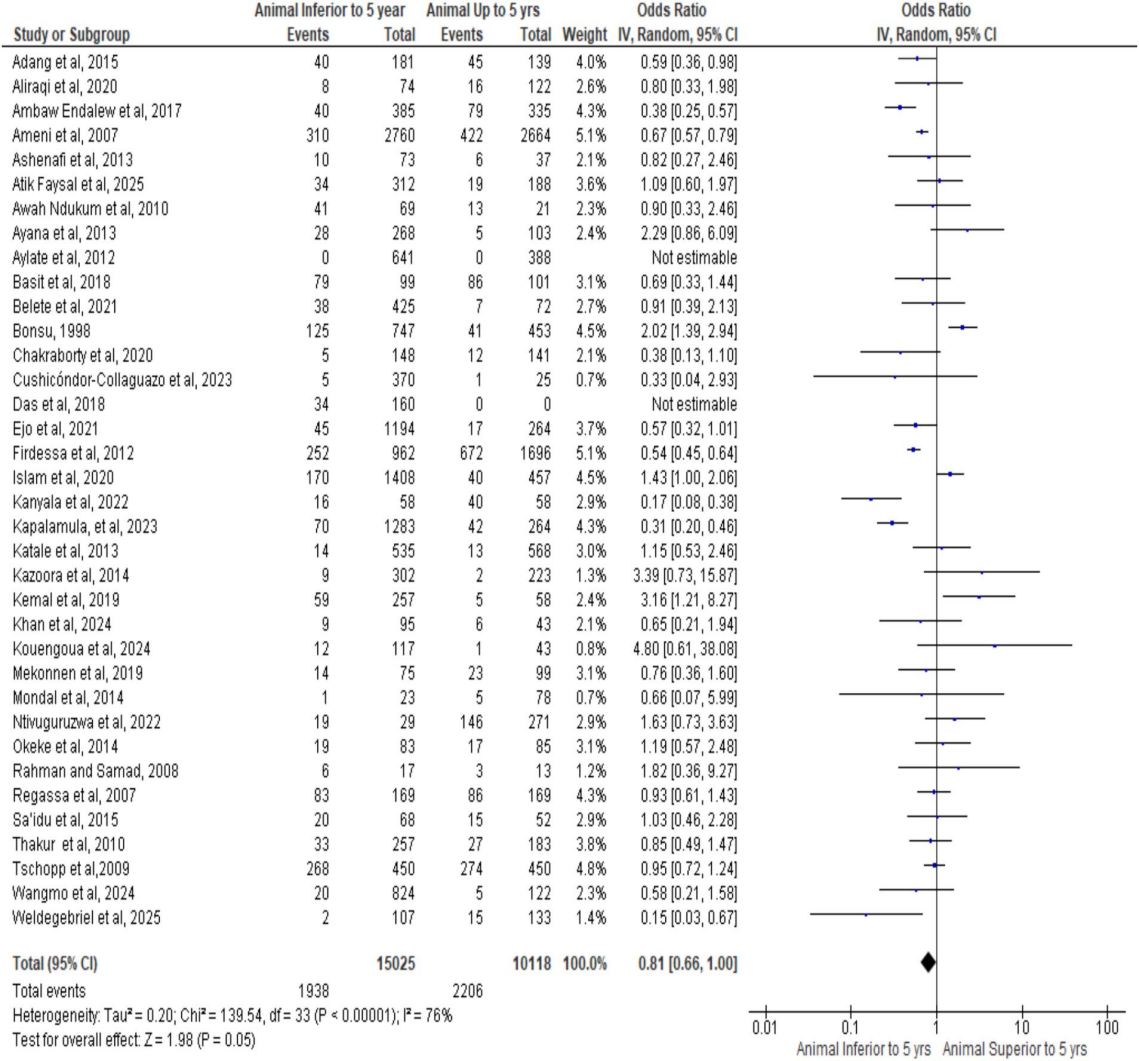
Forest plot of animals age.

Figure 8 forest plot shows pooled OR = 0.81 (95% CI: 0.66–1.00, *p* = 0.05), indicating borderline lower bTB risk in animals < 5 years. Substantial heterogeneity (I^2^ = 76%) reflects study and regional variability, with divergent individual estimates. Overall, results suggest age-related infection dynamics, emphasizing the need for age-stratified surveillance and tailored control strategies.

### 3.12 Publication of bias of included studies by age

**Figure 9:**
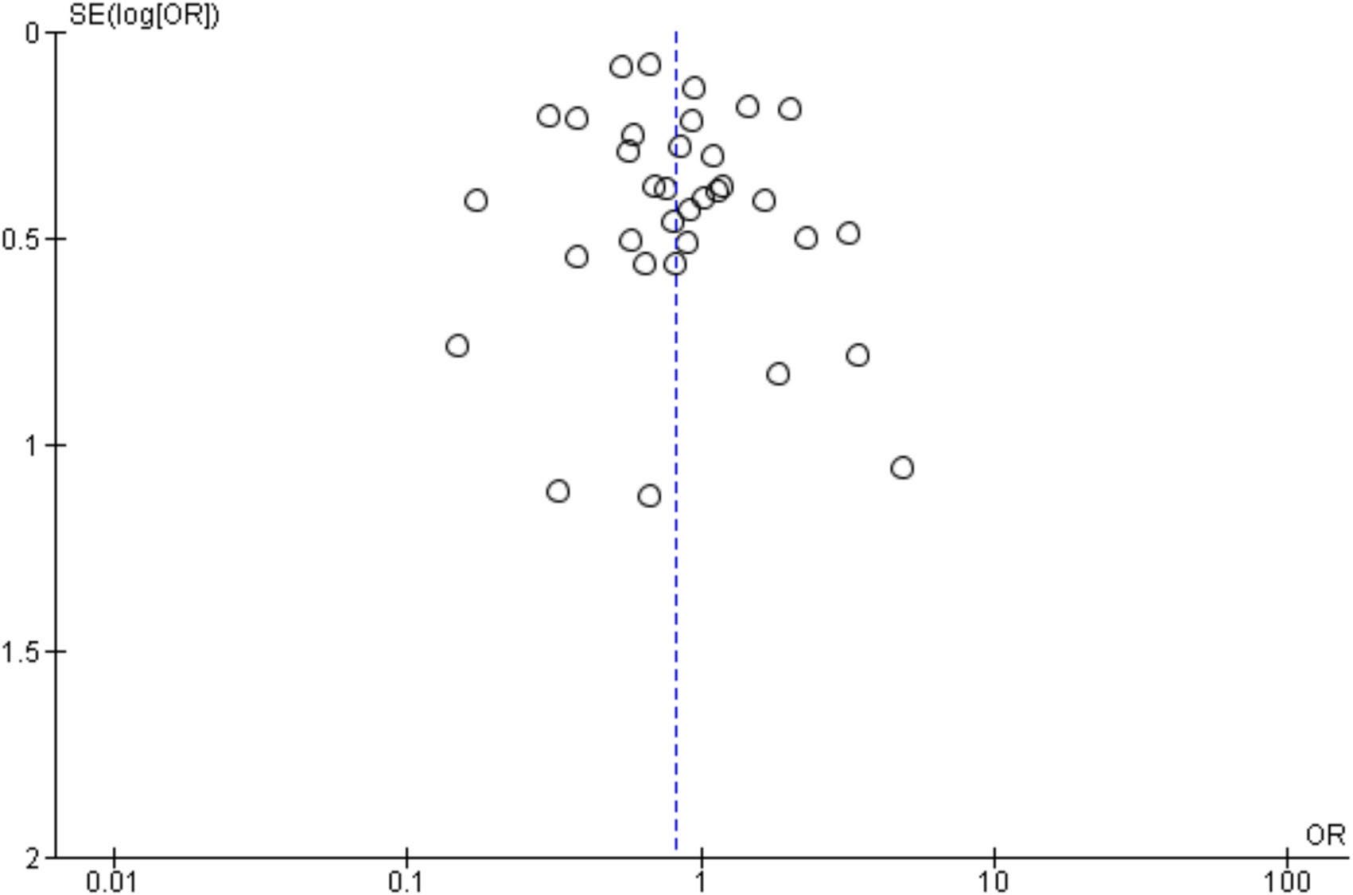
Funnel plot of animal by age.

Figure 9 funnel plot shows symmetrical distribution around OR = 0.81, with expected narrowing at low SE and widening at high SE, indicating minimal publication bias or small-study effects. Absence of asymmetry supports reliability despite moderate heterogeneity (I^2^ = 76%), confirming robustness of the age-related association and consistent methodological precision.

### 3.13 Meta-analysis of included studies by age in different continent

**Figure 10:**
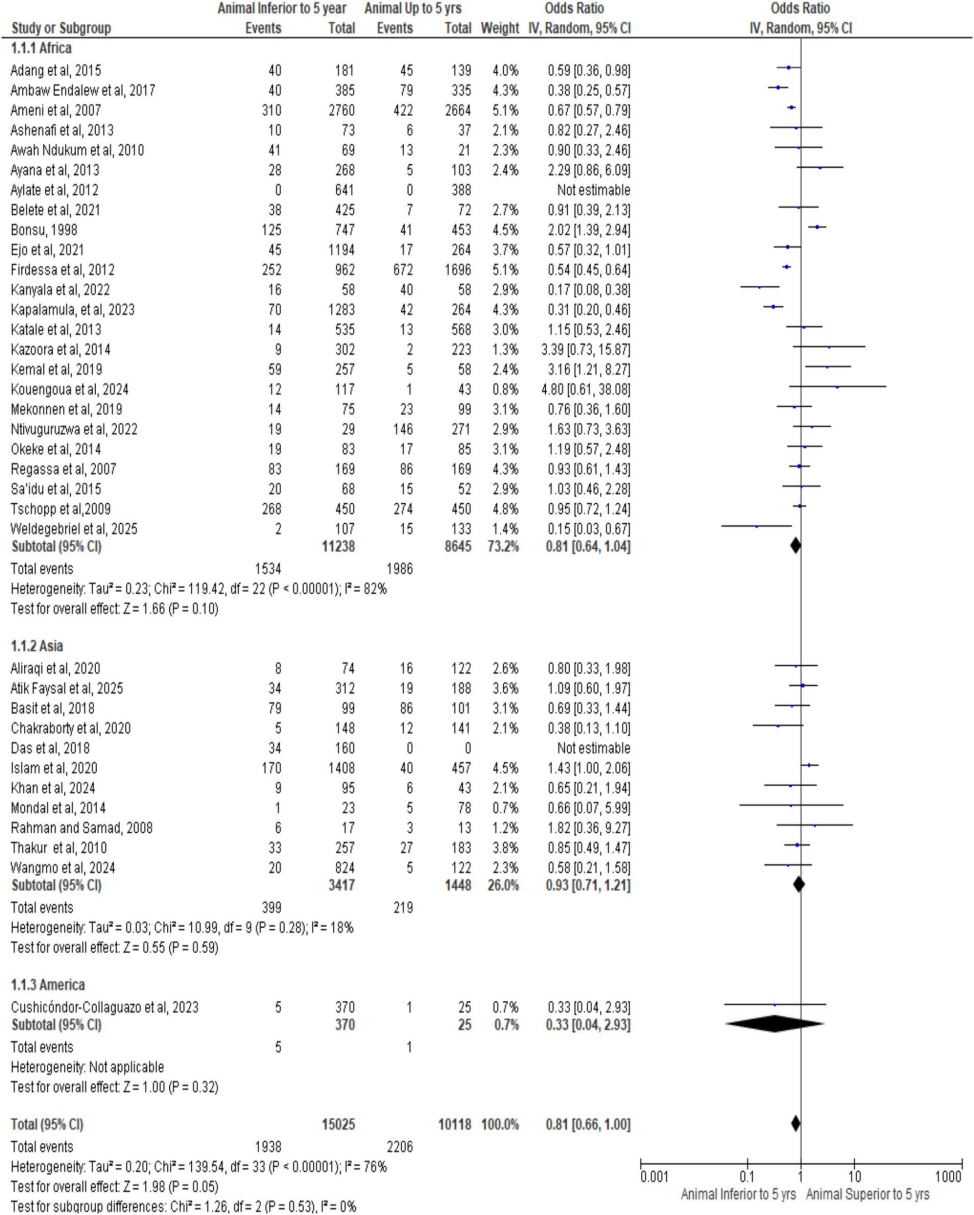
Forest plot of data by age per continent.

Figure 10 forest plot shows pooled OR = 0.81 (95% CI: 0.66–1.00, *p* = 0.05), indicating borderline lower bTB risk in animals <5 years. Subgroup analysis reveals heterogeneity: Africa (73.2% weight, OR = 0.81, I^2^ = 82%) suggests reduced risk; Asia shows neutral effect (OR = 0.93, I^2^ = 18%); America provides limited, imprecise data (OR = 0.33, wide CI). Overall heterogeneity (I^2^ = 76%) reflects methodological variability. Findings imply age-related infection dynamics, particularly in Africa, warranting age-stratified surveillance and standardized reporting.

### 3.14 Publication of bias of included studies by age in different regions

**Figure 11:**
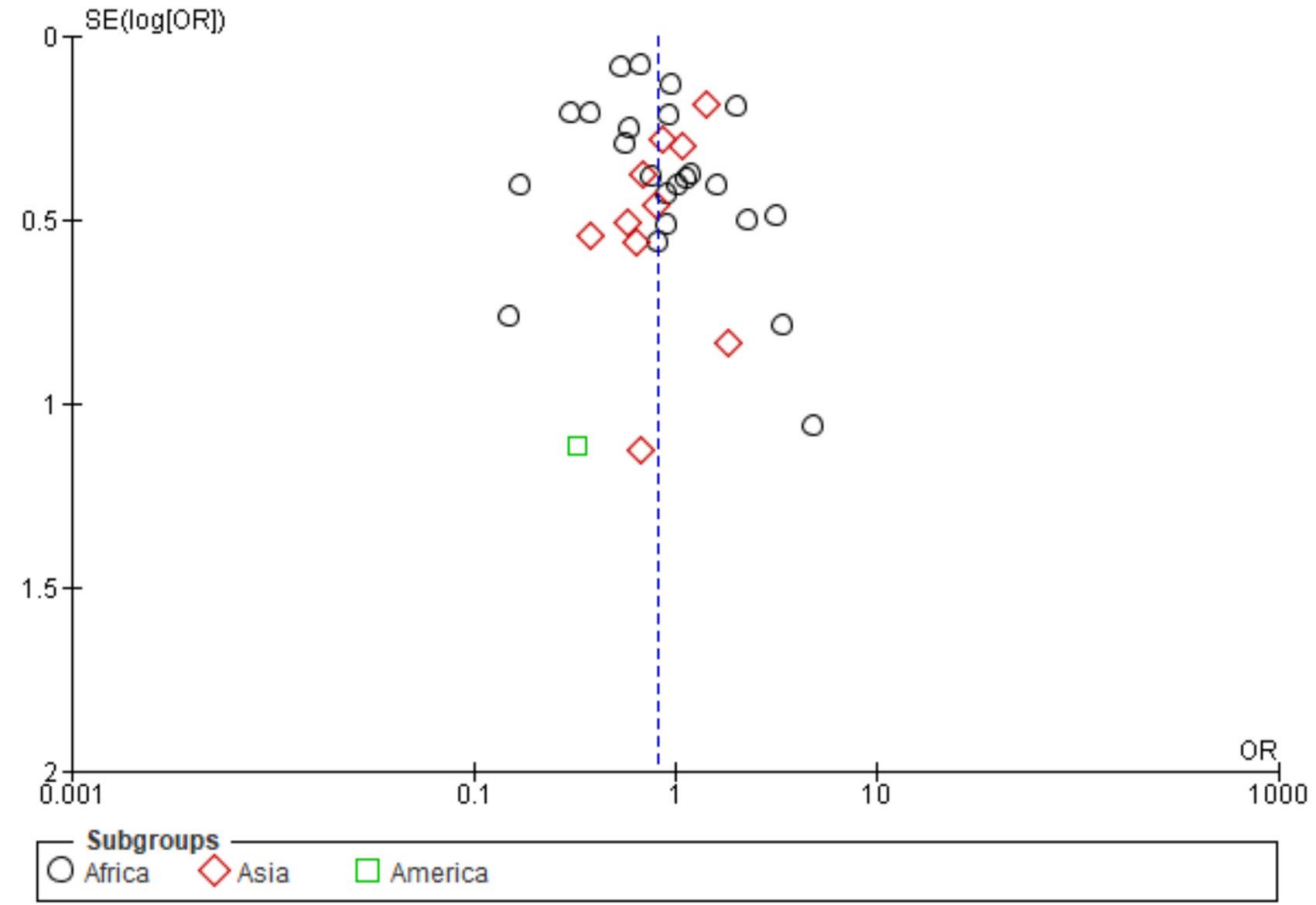
Funnel plot of animal age per continent.

Figure 11 funnel plot shows moderate asymmetry around OR = 1, suggesting possible reporting bias and heterogeneity. African studies dominate with tighter clustering and higher precision, while Asian and American data are more dispersed. Overall imbalance warrants cautious interpretation and underscores the need for standardized age-stratified surveillance and broader geographic representation.

### 3.15 Meta-analysis of included studies by age in different regions

**Figure 12:**
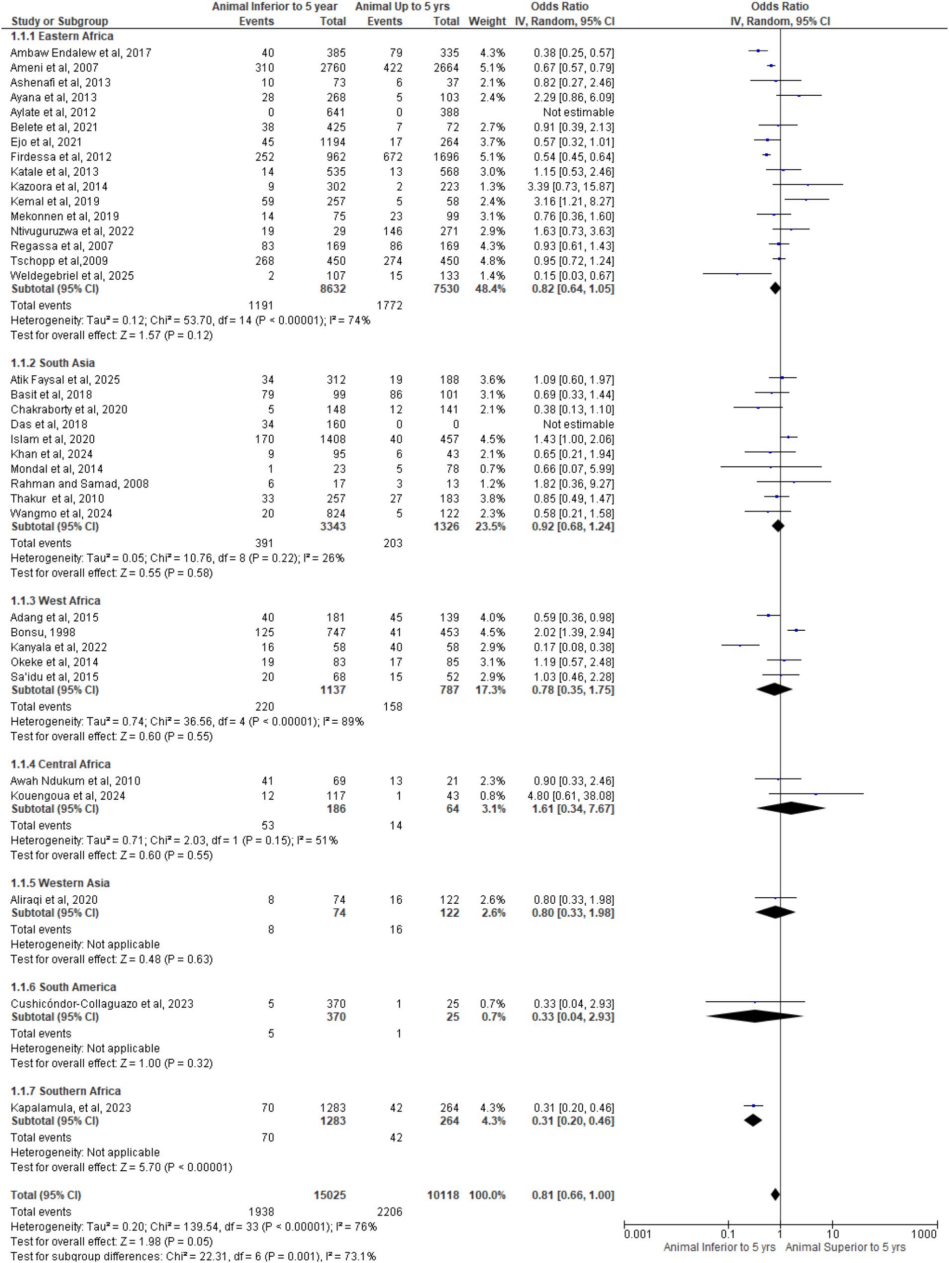
Forest plot of animals age by regions in the world.

Figure 12 forest plot shows pooled OR = 0.81 (95% CI: 0.66–1.00, *p* = 0.05), indicating borderline lower bTB risk in animals < 5 years. Subgroup results vary: Eastern Africa (OR = 0.82), West Africa (OR = 0.78), Southern Africa (OR = 0.31) suggest reduced odds, while South Asia (OR = 0.92) and Central Africa (OR = 1.61) show no consistent trend. High heterogeneity (I^2^ = 76%; Chi^2^ = 23.41, *p* = 0.001) reflects regional and methodological variability. Findings imply age-related infection dynamics, particularly in high-burden regions, modulated by local transmission and surveillance sensitivity.

### 3.16 Publication of bias of included studies by age in different regions

**Figure 13:**
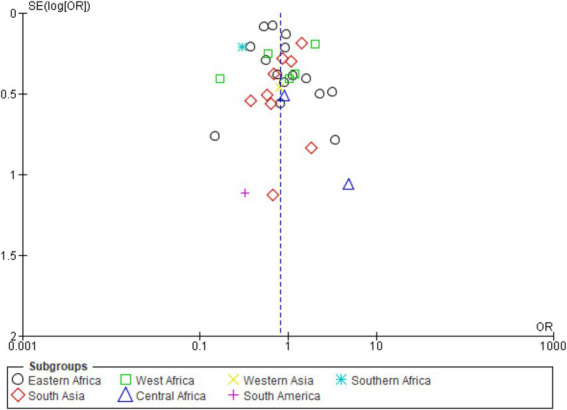
Funnel plot of animals age by regions in the world.

Figure 13 funnel plot shows near-symmetry around OR = 1, indicating minimal publication bias. Most subgroups (Eastern/Southern Africa, South Asia, West Africa) cluster at low SE, reflecting high precision. Absence of marked asymmetry supports pooled estimate (OR = 0.81), though heterogeneity (I^2^ = 76%) signals methodological variability. Findings confirm robustness while underscoring need for standardized age-stratified surveillance.

### 3.17 Comparative analysis of different cattle breeds

Table 7 shows breed-specific heterogeneity in bTB prevalence. Holsteins exhibit highest burden (30.1%, RR = 2.49), while indigenous zebus show lowest prevalence (9.1%, RR = 0.38), suggesting genetic resilience. Crossbreds present intermediate but variable rates (20.2%, 2.4–78.9%). Other breeds (Friesians 49.2%, White Fulani 33.2%, Landim 35.7%, Ankole 27.7%) are highly affected, whereas Jerseys (6.4%) and Boran (10.2%) remain less impacted. Extreme values in small samples indicate possible bias. Overall, exotic and crossbred cattle are disproportionately susceptible, underscoring the need for breed-tailored control strategies.

**Table 7:** Prevalence of bTB by breed.

Breed group	Number of studies	Total sample	Positive cases	Pooled prevalence (%)	95% CI	*P*-value	Risk ratio
Holsteins	9	4,601	1,384	30.08	28.76–31.41	0.020	2.49
Indigenous local zebus	13	10,585	961	9.08	8.53–9.63	0.007	0.38
Crosses (mixed breeds)	21	9,239	1,862	20.15	19.34–20.97	0.001	1.28
Gudali	2	55	10	18.18	7.99–28.38	0.243	1.01
Landim	1	468	167	35.68	31.34–40.02	0.000	2.02
White Fulani	3	286	95	33.22	27.76–38.98	0.063	1.86
Red Fulani	1	70	20	28.57	17.99–39.15	0.000	1.59
Red Bororo	2	132	39	29.55	21.76–37.33	0.190	1.64
Ankole	4	217	60	27.65	21.70–33.60	0.079	1.54
Friesian	6	378	186	49.21	44.17–54.25	0.052	1.39
Bonsmara	1	88	0	0.00	–	0.000	0.00
Jersey	2	189	12	6.35	2.87–9.83	0.196	0.35
Boran	1	216	22	10.19	7.07–16.21	0.000	0.56
Muturu	2	90	22	24.44	15.57–33.32	0.140	1.36

### 3.18 Spatio-temporal trends of bTB prevalence (1990–2025)

**Figure 14:**
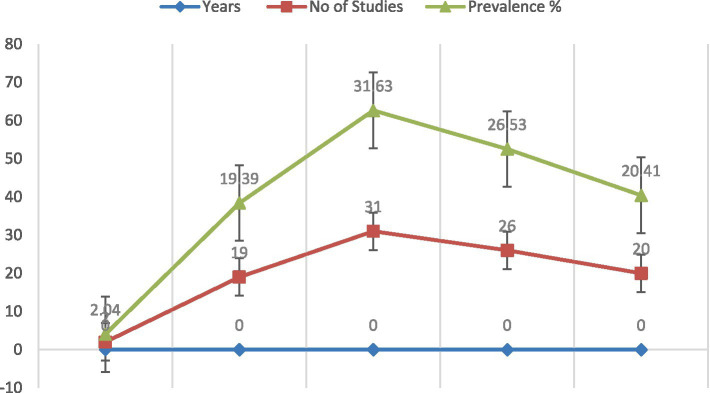
Spatio-temporal analysis of bTB prevalence.

Prevalence of bTB increased sharply from 1990 to 2015, peaking at 31.63%, then declined post-2015. Study volume followed a similar trajectory, suggesting intensified surveillance during peak years. The initial low prevalence (2.04%) may reflect underdiagnosis. Post-2015 decline (20.41%) indicates possible control efforts, though sustained transmission persists. Temporal clustering supports targeted interventions and longitudinal monitoring (Figure 14).

## 4 Discussion

The present meta-analysis provides a comprehensive and analytically grounded synthesis of bovine tuberculosis (bTB) epidemiology, integrating prevalence estimates with critical evaluation of methodological heterogeneity, diagnostic variability, and structural biases in surveillance systems. While the pooled animal-level prevalence (3.98%) and herd-level estimate (18.48%) suggest a relatively moderate global burden, the extremely high heterogeneity (I^2^ up to 94%) indicates that these summary measures should be interpreted with caution, as they likely reflect methodological inconsistencies rather than true epidemiological uniformity. Such heterogeneity arises from variations in study design, population structure, diagnostic tools, and regional surveillance intensity, reinforcing that pooled estimates represent composite indicators rather than biologically comparable metrics (20). The markedly higher herd-level prevalence compared to individual animal estimates further highlights the clustered nature of infection and underscores the epidemiological importance of herd-based surveillance in capturing transmission dynamics.

Geographical disparities observed across continents and regions further underscore the structural drivers of heterogeneity. The dominance of African (61.22%) and Asian (30.61%) datasets, coupled with significantly higher prevalence and risk ratios in Eastern Africa (RR = 10.51) and South Asia (RR = 6.41), likely reflects both a higher endemic burden and intensified targeted surveillance in these regions (21). Conversely, the limited representation and wide confidence intervals observed in Europe, Oceania, and the Americas suggest underrepresentation and reduced statistical power rather than true absence of disease (22). In many low- and middle-income countries, surveillance systems remain fragmented, often relying on abattoir-based inspections or targeted testing within specific production systems, which may either underestimate infection due to limited sensitivity or overestimate it through non-representative sampling (23). At the country level, the disproportionately high contribution of Ethiopia (34 studies; RR = 8.72; *p* = 0.004) highlights how pooled estimates may be driven by a few high-burden settings, introducing aggregation bias. These findings emphasize that surveillance data are not epidemiologically neutral but shaped by resource allocation, infrastructure, and national control policies, as previously reported (20).

Marked discrepancies in national prevalence estimates further illustrate this point. The global estimate reported here is lower than those documented in Pakistan (6.06%) (24) and India (7.3%) (24), yet higher than the figure reported in China (1.0%) (25). Conversely, substantially elevated prevalence rates have been described in Ghana (19%) (26) and South Africa (28%) (27).

A major source of variability identified in this study is the heterogeneity of diagnostic methods, which significantly influenced prevalence estimates (*p* = 0.015). Tuberculin skin tests (TST), particularly SICCT and CIDT, yielded the highest prevalence (42.86%; RR = 9.13), which may reflect their high sensitivity in field conditions but also their susceptibility to false positives due to cross-reactivity with environmental mycobacteria (24, 25). In contrast, molecular techniques (e.g., PCR, qPCR, and GeneXpert) showed lower prevalence (23.47%; RR = 5.24), increasingly reported in recent studies (28), offer improved specificity (reduced sensitivity in paucibacillary samples) and faster detection but remain constrained by cost, infrastructure, and technical expertise, limiting their widespread application in endemic regions (20). Culture methods, considered the gold standard, demonstrated relatively low prevalence (8.16%; RR ≈ 1), primarily due to their limited sensitivity, prolonged turnaround time, and dependence on viable organisms (29). Similarly, microscopy and meat inspection approaches exhibited poor diagnostic performance, consistent with their inability to detect subclinical infections. Immunological assays, including IFN-*γ* tests and serological methods, showed variable performance, largely influenced by host immune status and stage of infection. These findings highlight a fundamental diagnostic paradox: highly sensitive tests may overestimate prevalence, whereas highly specific tests may underestimate it (29–31). This diagnostic inconsistency is widely documented in recent literature (29–31) and remains a critical barrier to standardization and comparability of bTB surveillance data.

Specimen-related variability further contributes to diagnostic heterogeneity. The predominance of skin-based detection (RR = 8.86) reflects reliance on tuberculin testing, whereas blood and milk samples, commonly used in immunological and molecular assays, showed intermediate detection rates. Lower yields from tissue and nasal samples likely reflect sampling limitations and pathogen distribution (32). Importantly, inconsistencies between ante-mortem and post-mortem diagnostics—such as discordance between TST and culture or PCR—highlight the complex pathobiology of *Mycobacterium bovis* infection and the influence of host immune status. These findings corroborate previous reports on diagnostic discordance and cross-reactivity (32, 33), emphasizing the need for integrated, multi-test approaches. Similarly, the elevated prevalence in production and dairy systems may be driven by intensified surveillance and higher animal density rather than intrinsic differences in susceptibility (33).

The observed age-related and breed-associated differences also require cautious interpretation in light of heterogeneity. Higher prevalence in older animals (≥5 years) likely reflects cumulative exposure and chronic infection dynamics, however, the borderline statistical significance and substantial heterogeneity suggest that this association is context-dependent and influenced by local transmission dynamics and diagnostic sensitivity (34).

Breed-associated differences provide additional insight into host susceptibility, with higher prevalence observed in exotic breeds such as Holstein and Friesian cattle compared to indigenous zebus. While this finding aligns with recent literature suggesting genetic susceptibility, it is likely confounded by management practices, as exotic breeds are typically raised in intensive systems characterized by higher stocking density, production stress and increased transmission risk (35). Therefore, disentangling genetic predisposition from environmental and managerial factors remains essential for accurate risk attribution.

Importantly, this study highlights persistent structural weaknesses in bTB control, particularly in endemic regions. Inadequate implementation of test-and-slaughter policies, absence of compensation mechanisms, and limited integration between veterinary and public health sectors continue to hinder effective control. These challenges are further compounded by zoonotic risks associated with the consumption of unpasteurized milk and close human–animal interactions (36). Although temporal trends suggest a modest decline in prevalence after 2015, likely reflecting increased awareness and intervention efforts, sustained transmission indicates that current strategies remain insufficient.

Therefore, this study also highlights significant biases in surveillance data. The overrepresentation of certain regions (e.g., Eastern Africa) and underrepresentation of others introduces geographic bias, while the predominance of moderate-quality studies and limited number of “strong” studies (≈10%) raises concerns about internal validity (37). Although funnel plot analyses suggest low publication bias overall, the presence of small-study effects and regional asymmetry indicates that reporting bias cannot be entirely excluded (37, 38). Furthermore, differences in study objectives (production vs. slaughterhouse surveillance), sampling strategies, and diagnostic thresholds contribute to systematic bias across datasets.

From a One Health perspective, the persistence of bTB in endemic regions has important zoonotic implications, particularly in settings where unpasteurized milk consumption is common. Despite global efforts such as the End TB Strategy (39), the integration of veterinary and human health surveillance remains insufficient in many high-burden countries, limiting effective control of zoonotic transmission. In many developing countries, a substantial proportion of milk is marketed unpasteurized, increasing the risk of *M. bovis* transmission to humans. This concern is particularly salient in high-burden tuberculosis countries such as India (33). Recent studies continue to emphasize the need for coordinated, cross-sectoral approaches to bTB control, particularly in Africa and South Asia.

In general, this meta-analysis demonstrates that the global epidemiology of bTB is shaped by a complex interplay of diagnostic heterogeneity, surveillance biases, and contextual risk factors rather than uniform biological processes. The high degree of heterogeneity observed fundamentally limits the interpretability of pooled estimates and underscores the need for caution in cross-study comparisons. Addressing these challenges requires the harmonization of diagnostic protocols, strengthening of surveillance systems, and more balanced geographical representation of data. A coordinated One Health approach integrating veterinary and human health sectors will be essential to improve epidemiological accuracy and to design effective, evidence-based strategies for the control and eventual elimination of bovine tuberculosis.

## 5 Conclusion

This systematic review and meta-analysis highlight the global paucity of data on the prevalence of bovine tuberculosis (bTB) across different animal species and in several developing countries. Despite these gaps, evidence confirms the presence of *Mycobacterium bovis* on five continents, with an overall prevalence of 3.98%. Tuberculin-based skin tests emerged as the most frequently employed diagnostic approach, followed by molecular techniques, with skin and blood samples being the predominant specimen types. Prevalence varied markedly across countries and regions, with Ethiopia and Pakistan, as well as Eastern Africa and South Asia, showing the highest incidence, while East Asia, Western Asia (Middle East), South America, North America, Oceania, and parts of Europe reported the lowest. This geographical heterogeneity underscores the urgent need for regionally tailored collaborative strategies to control, eradicate, and prevent bTB in livestock populations. Such strategies must account for local risk factors and epidemiological contexts to effectively limit transmission and mitigate associated hazards.

## Data Availability

The datasets generated and/or analyzed during the current study are available from the corresponding author upon reasonable request.
