# Supplementary material for: Global prevalence and risk factors of bovine tuberculosis in cattle: a systematic review and meta-analysis
Source: Front Vet Sci. 2026 Apr 22;13:1820176. doi: 10.3389/fvets.2026.1820176 (PMC13143542; doi:10.3389/fvets.2026.1820176)
Supplement: Supplementary file 1 [file Table_1.DOCX]

**Supplementary Material S1: Detailed Search Strategy**

A comprehensive and systematic literature search was conducted to identify all relevant studies reporting the prevalence, risk factors, and diagnostic methods of bovine tuberculosis (bTB) in cattle worldwide. The search strategy was developed using a combination of controlled vocabulary (e.g., MeSH terms) and free-text keywords to maximize sensitivity and specificity.

**1. Core Search String (General Boolean Strategy)**

The following Boolean search expression was used as the foundation:

("Mycobacterium bovis" OR "bovine tuberculosis" OR "bTB" OR "bovine TB")
AND
("prevalence" OR "seroprevalence" OR "epidemiology" OR "occurrence" OR "incidence")
AND
("risk factors" OR "determinants" OR "associated factors" OR "predictors")
AND
("diagnosis" OR "detection" OR "diagnostic methods" OR "screening" OR "testing" OR "tuberculin skin test" OR "TST" OR "PCR" OR "polymerase chain reaction" OR "culture" OR "ELISA" OR "interferon gamma assay")
AND
("cattle" OR "bovine" OR "Bos taurus" OR "Bos indicus")
AND
("Africa" OR "Asia" OR "Europe" OR "America" OR "Oceania" OR "global" OR "worldwide")

**2. Database-Specific Search Strategies**

**2.1. PubMed / MEDLINE (with MeSH terms)**

("Mycobacterium bovis"[Mesh] OR "bovine tuberculosis"[Title/Abstract] OR "bTB"[Title/Abstract])
AND
("Prevalence"[Mesh] OR "Epidemiology"[Mesh] OR prevalence[Title/Abstract] OR seroprevalence[Title/Abstract] OR incidence[Title/Abstract])
AND
("Risk Factors"[Mesh] OR "risk factors"[Title/Abstract] OR determinants[Title/Abstract] OR predictors[Title/Abstract])
AND
("Diagnosis"[Mesh] OR diagnosis[Title/Abstract] OR detection[Title/Abstract] OR "diagnostic methods"[Title/Abstract] OR "tuberculin test"[Title/Abstract] OR PCR[Title/Abstract] OR culture[Title/Abstract] OR ELISA[Title/Abstract])
AND
("Cattle"[Mesh] OR cattle[Title/Abstract] OR bovine[Title/Abstract] OR "Bos taurus"[Title/Abstract] OR "Bos indicus"[Title/Abstract])

**2.2. Scopus**

TITLE-ABS-KEY ( "Mycobacterium bovis" OR "bovine tuberculosis" OR "bTB" )
AND
TITLE-ABS-KEY ( prevalence OR seroprevalence OR epidemiology OR incidence )
AND
TITLE-ABS-KEY ( "risk factors" OR determinants OR predictors )
AND
TITLE-ABS-KEY ( diagnosis OR detection OR "diagnostic methods" OR "tuberculin test" OR PCR OR culture OR ELISA )
AND
TITLE-ABS-KEY ( cattle OR bovine OR "Bos taurus" OR "Bos indicus" )
AND
TITLE-ABS-KEY ( Africa OR Asia OR Europe OR America OR Oceania OR global OR worldwide )

**2.3. Web of Science**

TS = ( "Mycobacterium bovis" OR "bovine tuberculosis" OR "bTB" )
AND TS = ( prevalence OR seroprevalence OR epidemiology OR incidence )
AND TS = ( "risk factors" OR determinants OR predictors )
AND TS = ( diagnosis OR detection OR "diagnostic methods" OR "tuberculin test" OR PCR OR culture OR ELISA )
AND TS = ( cattle OR bovine OR "Bos taurus" OR "Bos indicus" )
AND TS = ( Africa OR Asia OR Europe OR America OR Oceania OR global OR worldwide )

**2.4. Google Scholar (simplified strategy)**

"Mycobacterium bovis" OR "bovine tuberculosis"
AND cattle OR bovine
AND prevalence OR epidemiology
AND "risk factors" OR diagnosis OR detection

**3. Additional Search Procedures**

- Manual screening of reference lists of included articles
- Screening of review articles for additional eligible studies
- Inclusion of studies published between **1990 and 2025**
- Restriction to **English and French languages** (acknowledged as a potential limitation)

**4. Search Strategy Justification**

This search strategy was designed to:

- Capture **all relevant epidemiological studies** on bTB
- Include **multiple synonyms and diagnostic approaches**
- Ensure **broad geographic coverage (global scope)**
- Balance **sensitivity (comprehensive retrieval)** and **specificity (relevance)**
